# Co-chaperone p23 Regulates *C*. *elegans* Lifespan in Response to Temperature

**DOI:** 10.1371/journal.pgen.1005023

**Published:** 2015-04-01

**Authors:** Makoto Horikawa, Surojit Sural, Ao-Lin Hsu, Adam Antebi

**Affiliations:** 1 Max Planck Institute for Biology of Ageing, Cologne, Germany; 2 University of Michigan, Department of Internal Medicine, Division of Geriatric and Palliative Medicine, Ann Arbor, Michigan, United States of America; 3 University of Michigan, Department of Molecular and Integrative Physiology, Ann Arbor, Michigan, United States of America; 4 Cologne Excellence Cluster on Cellular Stress Responses in Ageing Associated Diseases (CECAD), University of Cologne, Cologne, Germany; 5 Department of Molecular and Cellular Biology, Huffington Center on Ageing, Baylor College of Medicine, Houston, Texas, United States of America; Stanford University Medical Center, UNITED STATES

## Abstract

Temperature potently modulates various physiologic processes including organismal motility, growth rate, reproduction, and ageing. In ectotherms, longevity varies inversely with temperature, with animals living shorter at higher temperatures. Thermal effects on lifespan and other processes are ascribed to passive changes in metabolic rate, but recent evidence also suggests a regulated process. Here, we demonstrate that in response to temperature, *daf-41*/ZC395.10, the *C*. *elegans* homolog of p23 co-chaperone/prostaglandin E synthase-3, governs entry into the long-lived dauer diapause and regulates adult lifespan. *daf-41* deletion triggers constitutive entry into the dauer diapause at elevated temperature dependent on neurosensory machinery (*daf-10/IFT122*), insulin/IGF-1 signaling (*daf-16*/FOXO), and steroidal signaling (*daf-12*/FXR). Surprisingly, *daf*-41 mutation alters the longevity response to temperature, living longer than wild-type at 25°C but shorter than wild-type at 15°C. Longevity phenotypes at 25°C work through *daf-16*/FOXO and heat shock factor *hsf-1*, while short lived phenotypes converge on *daf-16*/FOXO and depend on the *daf-12*/FXR steroid receptor. Correlatively *daf-41* affected expression of DAF-16 and HSF-1 target genes at high temperature, and nuclear extracts from *daf-41* animals showed increased occupancy of the heat shock response element. Our studies suggest that *daf-41*/p23 modulates key transcriptional changes in longevity pathways in response to temperature.

## Author Summary

Temperature is a critical environmental factor that affects ageing in both cold-blooded and warm-blooded species. In invertebrate animals, lifespan varies inversely with temperature, with higher temperature resulting in faster development but shorter lifespan. This phenomenon has been usually attributed to passive changes in metabolic rate, but recent work suggests that this process is regulated. In this study, we identify the co-chaperone protein p23 in the nematode *C*. *elegans* as an important modulator of longevity in response to temperature. Co-chaperones bind to client proteins to assist in their folding or stabilize their shape, thereby regulating their activity. Remarkably, deletion of p23 results in animals that are long lived at high temperatures and short lived at low temperatures relative to normal wild type animals. Our experiments indicate that p23 regulates lifespan through the neurosensory apparatus. These in turn impinge on key longevity regulators that mediate the transcriptional outputs of insulin/IGF, heat shock response and steroidal signaling. These studies suggest that complexes formed by p23 play a central role in regulating longevity in response to temperature.

## Introduction

Temperature dramatically impacts the lifespan of ectotherms, with lower temperatures typically extending and higher temperatures shortening life [1–3]. The conventional view is that temperature passively affects the rate of chemical reactions and metabolism, thereby influencing species longevity. An emerging body of evidence, however, indicates that changes in longevity in response to temperature also reflect a regulated process entailing important organismal adaptations [4–6].

Like other ectotherms, the nematode *Caenorhabditis elegans* shows clear temperature dependent influences on development and lifespan [2]. At the normal cultivation temperature (20°C), animals typically live three weeks. At low temperature (15°C) they live approximately ten days longer, and at high temperature (25°C) ten days shorter. A handful of identified loci have been recently shown to mediate changes in lifespan in response to temperature. At warm temperatures, signaling from thermotaxis neurons promotes normal lifespan, and activates steroid hormone signaling to maintain longevity [4]. Animals compromised in thermotaxis genes or steroid hormone production display shortened life. In addition, animals will mount a heat shock response when exposed to acute heat stress, which helps preserve organismal viability. Thermotaxis neurons regulate the organismal heat shock response through the heat shock transcription factor HSF-1 [7]. Overexpression of *hsf-1* and downstream chaperones can extend lifespan [8,9]. In contrast, longevity at cool temperatures requires the cold sensitive TRPA-1 channel [5], which works through Ca^2+^ signaling, protein kinase-C PKC-2, and serum and glucocorticoid kinase SGK-1 to activate the forkhead transcription factor DAF-16/FOXO, a crucial regulator of longevity. Evidently the mechanisms governing longevity at low or high temperature appear somewhat different and few components affect both [4].

Temperature effects on organismal longevity are not limited to ectotherms. Notably, temperature sensing neurons involved in homeostatic control of core body temperature affect murine lifespan [6]. Higher temperatures in these neurons trigger a lowering of the core body temperature and correlate with extended lifespan. Several long-lived mouse models including the Ames Dwarf, Growth Hormone Receptor knockout, and FGF21 transgenic mice have associated a lower core body temperature reminiscent of nutrient induced torpor [10,11]. Furthermore cold exposure extends lifespan and suppresses tumorigenesis in rats [12]. These studies suggest an intimate but relatively unexplored relationship between nutrient and thermal sensing, metabolism and longevity.

At the cellular level, chaperones and co-chaperones facilitate protein folding and assembly, often in response to thermal stress [13,14]. One such co-chaperone is p23 [15]. p23 complexes with the HSP90 chaperone and inhibits its ATPase activity [16–18], thereby stabilizing association with client proteins such as steroid receptor transcription factors, heat shock factor, and others [19–25]. These interactions play an important role in regulating transcriptional events. p23 also displays HSP90 independent chaperone-like activity [26], and is implicated in various cellular functions [27]. Additionally the protein reportedly harbors prostaglandin E2 synthase (PGS) activity in vitro [28], although this is debated [29]. Interestingly, p23 upregulation has also been implicated in tumorigenesis presumably through its interactions with HSF1, steroid receptors or growth regulated kinases [30]. Indeed, several anti-cancer drugs being developed target chaperones and co-chaperones, highlighting the clinical importance of these pathways. p23 knockout mice reveal an early peri-natal lethal phenotype, with defects in lung and skin development, but its organismal roles remain largely unknown [29,31].

Here, we used *C*. *elegans* to explore the role of p23 function in metazoan biology. We found that *daf-41*, the *C*. *elegans* homolog of co-chaperone p23, has a novel role in regulation of lifespan at both high and low temperatures, as well as in the formation of the long lived dauer stage. Remarkably, *daf-41* mutants provoked longevity at warm temperatures, but short lived phenotypes at cold temperatures, thus equalizing the temperature response. *daf-41* interacted with insulin signaling, heat shock factor, and steroidal signaling to regulate lifespan by distinct mechanisms at different temperatures. Our findings implicate *daf-41* as a central player in the thermal regulation of longevity.

## Results

### *daf-41/*p23 is a novel regulator of dauer formation

**Fig 1:**
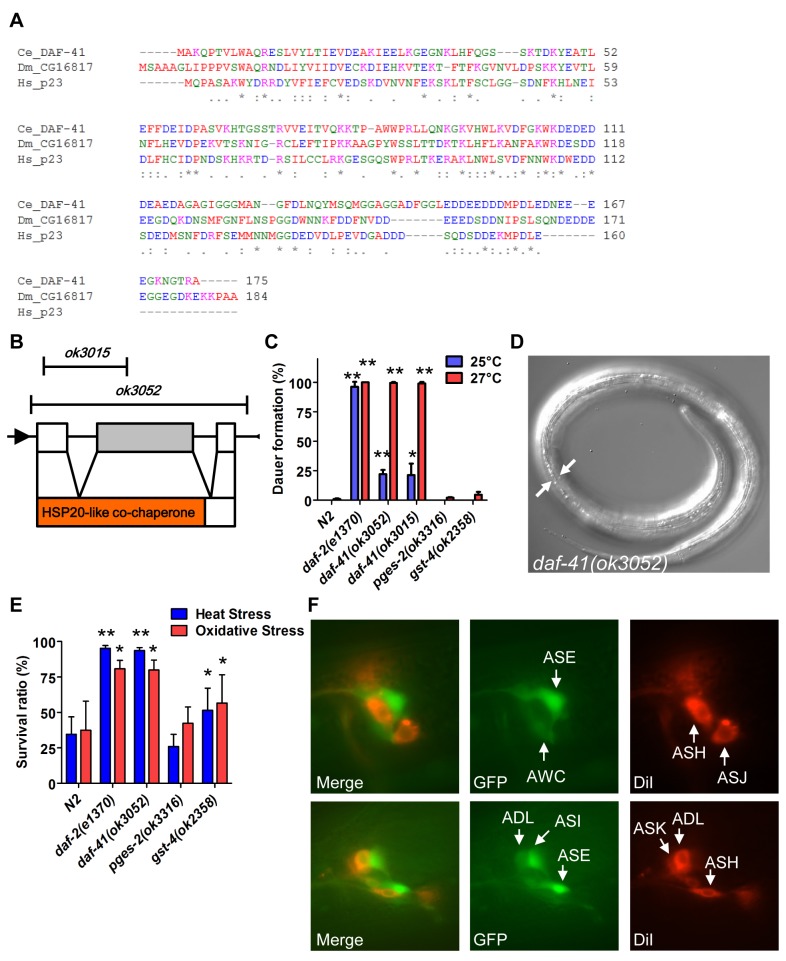
*daf-41*/ZC395.10 regulates dauer formation and stress resistance. (A) An alignment of protein sequences between *C*. *elegans* DAF-41, *D*. *melanogaster* CG16817 and *Homo sapiens* p23*/PTGES3*. The similarity between DAF-41 and p23*/PTGES3* is 44.6%. (B) Schematic illustration of the *daf-41*, and deletion alleles of *ok3015* and *ok3052*. Black arrows indicate the direction of transcription. Red area indicates HSP20-like co-chaperone domain. (C) *daf-41* mutants constitutively formed dauer larvae (Daf-c) weakly at 25°C and strongly at 27°C. (D) Dauer alae of *daf-41(ok3052)* animals grown at 27°C are indicated by the white arrows. (E) *daf-41(ok3052)* worms were resistant for oxidative stress (20mM of H_2_O_2,_ 2.5 hrs) and heat stress (35°C, 8 hrs). *gst-4(ok2358)* worms were also slightly stress tolerant. (F) *daf-41p*::*gfp* (i.e. *dpy-5(e907); sEx10796 [rCes daf-41*::*gfp + pCeh361])* worms were labeled with DiI and photos taken at the young adult stage. Patterns of gene expression of *daf-41p*::*gfp* (green), DiI (red), and merged figures are shown, with arrows indicating individual neurons.

DAF-41*/*ZC395.10 is the sole *C*. *elegans* homolog of co-chaperone p23/cytosolic prostaglandin E synthase-3. DAF-41/p23 is broadly conserved in evolution, with approximately 45% peptide similarity to the human homolog (Fig. 1A). The protein contains an N-terminal HSP20-like co-chaperone domain, implicated in HSP90 binding, and a C-terminal region, implicated in intrinsic chaperone activity (Fig. 1B). *daf-41(ok3052)* is a deletion allele and presumptive null that removes co-chaperone and chaperone domains; *daf-41(ok3015)* harbors an in-frame deletion of the co-chaperone domain, leaving the chaperone domain intact (Fig. 1B).

Under conditions of food scarcity, overcrowding and elevated temperature, *C*. *elegans* larvae enter the dauer diapause, a stage specialized for survival and dispersal. We found that both *daf-41(ok3052)* and *daf-41(ok3015)* alleles were constitutively prone to form dauer larvae (Daf-c phenotype) at elevated temperature, yielding approximately 25% dauer larvae at 25°C, and nearly 100% dauer larvae at 27°C (Fig. 1C-D). By contrast, mutants containing deletions of other putative prostaglandin synthase homologs, *pges-2/*mPGES2, *gst-4/*PGDS, had little observable dauer phenotype at these temperatures. By inference, the co-chaperone function of *daf-41* may be more important for Daf-c phenotypes.

Many Daf-c loci in the dauer signaling pathways, such as the *daf-2*/Insulin receptor (InsR) mutant, provoke resistance to various forms of stress [32,33]. Similarly *daf-41* mutants displayed resistance to oxidative stress induced by H_2_O_2_ challenge comparable in strength to *daf-2*/InsR mutants (Fig. 1E). *daf-41* mutants exposed to heat stress at 35°C were also significantly resistant (Fig. 1E). Other PGS mutants had little effect on oxidative and heat resistance. Altogether these results demonstrate that *daf-41* is a novel Daf-c gene involved in stress tolerance.

To clarify *daf-41* function, we examined its expression pattern. A promoter fusion to *gfp*, *daf-41p*::*gfp*, revealed expression most prominently in anterior and posterior neurons (Fig. 1F) including amphids (e.g. ASE, AWC, ASI, ADL) and phasmid sensory neurons, as well as peripheral neurons and ventral cord motorneurons. We also observed strong expression in body wall muscle and pharynx, as well as occasional expression in vulva, seam and intestine (S1A-B Fig).

### *daf-41* regulates dauer formation via insulin/IGF, and steroidal signaling

**Fig 2:**
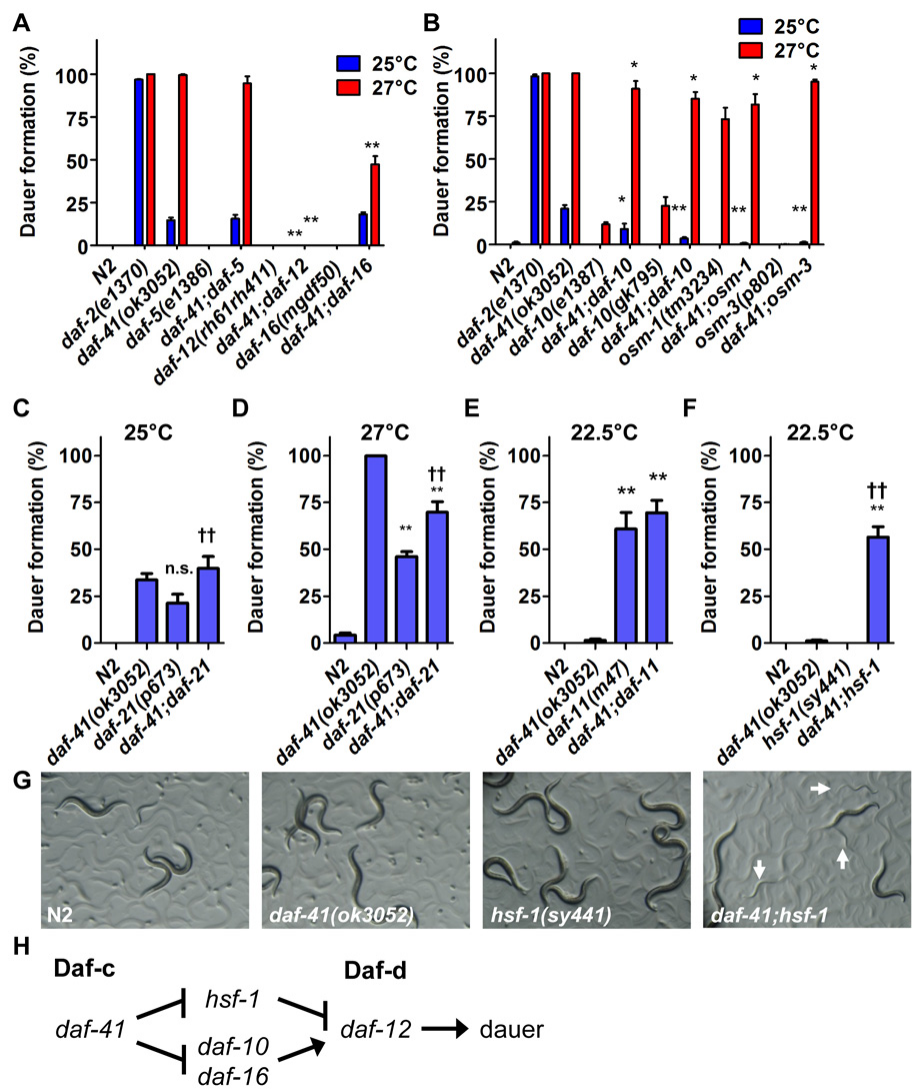
Genetic interactions of *daf-41* with dauer signaling pathways. (A) *daf-41(ok3052)* Daf-c phenotypes were partially suppressed in *af-16(mgDf50)* and completely suppressed in *daf-12(rh61rh411)* backgrounds. (B) *daf-41(ok3052)* Daf-c phenotypes were suppressed in various chemotaxis mutant backgrounds. (C-D) *daf-21(p673)* had no additive effect on Daf-c phenotypes at 25°C, and modestly reduced dauer formation at 27°C in the *daf-41(ok3052)* background. (E) *daf-11(m47)* had no additive effect on dauer formation in the *daf-41(ok3052)* background at 22.5°C. (F) *hsf-1(sy441)* strongly enhanced dauer formation of *daf-41(ok3052)* at 22.5°C. (G) Cultures of *daf-41(ok3052)*, *hsf-1(sy441) and daf-41;hsf-1* are shown grown at 22.5°C. White arrows indicate dauer larvae. All error bars indicate S.D. *, p<0.05; **, p<0.01 versus *daf-41(ok3052);* ††, p<0.01 versus *daf-21(p673)A* or *hsf-1(sy441)* by t-test. (H) *daf-41* regulates dauer formation via *daf-10*, *daf-12* and *daf-16* similar to *daf-21*. However, *hsf-1* suppresses dauer formation in *daf-21* but not *daf-41*. Note that the model reflects genetic interactions, not necessarily direct biochemical interactions.

In the dauer signaling pathways, environmental cues are detected by the neurosensory apparatus, and integrated by cGMP, TGF-β and insulin/IGF signaling. Ultimately these pathways converge on steroidal signaling to mediate the choice between arrest at the dauer diapause or continuous development to reproductive adult [34]. To understand where *daf-41* acts in the dauer signaling pathways, we performed genetic epistasis experiments for dauer formation at 27°C. We first combined *daf-41* Daf-c mutations with dauer formation defective (Daf-d) mutations in TGF-β signaling (*daf-5/*Ski), insulin/IGF signaling (*daf-16/*FOXO), and steroidal signaling (*daf-12/*FXR) [35–39]. As expected, null mutation of the steroid receptor, *daf-12*—a master regulator of dauer formation—completely suppressed *daf-41* Daf-c phenotypes. Mutation of *daf-16* partially suppressed *daf-41* Daf-c phenotypes, while *daf-5* mutation had little or no effect (Fig. 2A). At 25°C, *daf-41* Daf-c phenotypes were also suppressed by *daf-12*. These experiments reveal that *daf-41* acts upstream of *daf-16*, and *daf-12*, and in parallel to *daf-5*, to prevent dauer formation, resembling loci acting early in the dauer signaling pathways (Fig. 2H) [40].

### *daf-41* affects dauer formation and chemotaxis through neurosensory signaling

We next analyzed interactions between *daf-41* and neurosensory machineries of thermotaxis and chemotaxis, which variously affect dauer formation. We made double mutants with neurosensory transduction mutants (*daf-10/*IFT122, *osm-1* and *osm-3)*, which are Daf-d at 25°C and often Daf-c at 27°C [41,42]. We also examined thermotaxis mutants (*pkc-1*, *ttx-3*) that modulate dauer formation dependent on temperature and signaling pathway (e.g. *ttx-3* suppresses *daf-7*/TGF-β Daf-c phenotype at 25°C, but enhances it at 15°C) [43,44].

We found that mutations in the chemotaxis genes, *daf-10*, *osm-1* and *osm-3*, significantly suppressed Daf-c phenotypes of *daf-41(ok3052)* at 25°C and partially at 27°C (Fig. 2B). (Precedence for weaker Daf-c mutants suppressing stronger Daf-c mutants has been seen previously [45]). Consistent with a role proximal to the chemotaxis machinery, *daf-41(ok3052)* worms were chemotaxis defective for isoamylalcohol, benzaldehyde and 2,4,5-trimethylthiazoline (S2A Fig). By contrast, the thermotaxis loci did not appreciably affect *daf-41(ok3052)* Daf-c phenotypes at the examined temperatures (S3A-B Fig), although *pkc-1* had a minor effect at 27°C. Thus *daf-41* might work closely to chemotaxis loci and downstream or parallel to the thermotaxis loci.

Neurosensory cilia normally contact the environment through sensilla in head and tail, and typically fill with the lipophilic dye DiI. Mutants with defective neurosensory cilia structure, including *daf-10* and *osm-3* fail to fill with DiI because dendritic endings are not exposed [46]. We found that *daf-41* null mutants had normal DiI filling similar to wild type worms (S2B Fig). Altogether these results show that *daf-41* affects chemotaxis function, but not sensory cilia structure.

### Interactions with HSP90 and cGMP signaling

In its capacity as co-chaperone, p23 is known to complex with HSP90 [16–18,47]. The *C*. *elegans* homolog of HSP90, *daf-21*, functions early in the dauer signaling pathways at the level of chemosensory processing, similar to *daf-41*. *daf-21(p673)* is a weak gain-of-function (*gf*) amino acid substitution that renders animals Daf-c [48]. Genetic epistasis studies show that Daf-c phenotypes are suppressed by *daf-10* and other chemosensory mutants [40]. cGMP signaling represents another branch of the dauer signaling pathways that works at a similar level as *daf-21*. Mutations in the *daf-11*/transmembrane guanylyl cyclase provoke Daf-c phenotypes, which are similarly suppressed by chemosensory mutants [40,48]. Finally both *daf-21* and *daf-11* exhibit chemosensory deficits [49].

Because *daf-41*/p23 may regulate dauer entry through the chemosensory axis, we sought to dissect genetic interactions between *daf-41*, *daf-21* and *daf-11*. Although both *daf-41(ok3052)* and *daf-21(p673)* yielded Daf-c phenotypes, *daf-41;daf-21* worms did not show synthetic enhancement of dauer formation at 25°C (Fig. 2C). Furthermore, *daf-41* phenotypes were weakly suppressed by *daf-21* mutation at 27°C (conversely *daf-21* phenotypes were weakly enhanced by *daf-41*) (Fig. 2D). Likewise the *daf-41(ok3052)* null allele had little effect on dauer formation of *daf-11(m47)* at 22.5°C, where *daf-11’s* Daf-c phenotypes were partially penetrant (Fig. 2E). Whereas mutants in independent pathways typically give strong synergy, the modest interactions observed above suggest that *daf-41* could work in a proximal or overlapping pathway with *daf-21* and *daf-11*.

In mammals, HSP90 complexes are known to negatively regulate the activity of the heat shock transcription factor HSF1 [23,24]. Recently, it was reported that the *hsf-1(sy441)* missense mutation suppresses the Daf-c phenotypes of *daf-21* and *daf-11* at 23°C [50], suggesting that dauer formation depends upon active *hsf-1(+)*. We therefore analyzed genetic interactions between *hsf-1* and *daf-41* around this temperature. Surprisingly, instead of suppression, *hsf-1(sy441)* enhanced *daf-41* Daf-c phenotypes at 22.5°C (Fig. 2F). Similarly the egg laying defect of *hsf-1(sy441)* was strikingly enhanced in the *daf-41* mutant background (S4 Fig). These synthetic interactions suggest that *daf-41* and *hsf-1* could work closely together, or identify parallel pathways converging on the same process (Fig. 2H).

### *daf-41* regulates longevity in response to temperature

**Fig 3:**
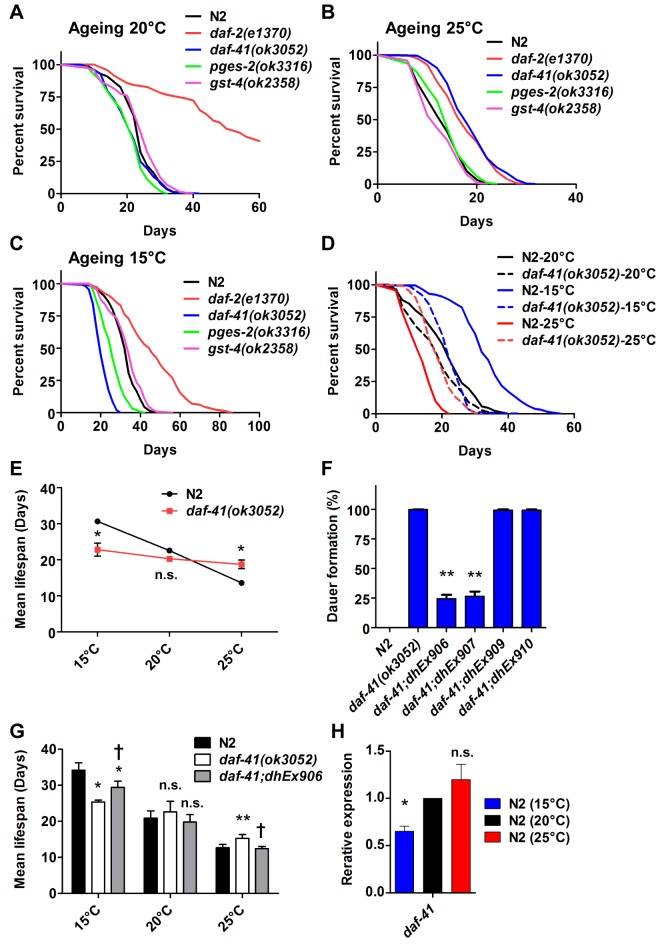
*daf-41* mutants extend lifespan at elevated temperatures and shorten life span at lower temperatures. (A) *daf-41(ok3052)* mutant animals have a lifespan similar to wild type N2 worms at 20°C (B) *daf-41(ok3052)* animals showed extended lifespan at 25°C (C) *daf-41(ok3052)* animals showed reduced lifespan at 15°C. (D) Lifespan curves of N2 and *daf-41(ok3052)* at different temperatures were plotted onto the same graph as indicated. (E) Mean lifespan of 3 individual experiments were plotted. Error bars, S.D. *, p<0.05; n.s., no significant difference versus N2 by t-test. (F) *daf-41(+)* transgenes rescued Daf-c phenotypes of *daf-41(ok3052)* at 27°C. *dhEx906* and *dhEx907* are independent *daf-41(+)* transgenic lines. *dhEx909* and *dhEx910* are *pges-2(+)* transgenes under control of *daf-41* 5’ and 3’ regulatory elements. Error bars, S.D.; **, p<0.001 versus non-transgenic worms by t-test. (G) Mean lifespan from 3 individual experiments at 15°C, 20°C and 25°C were plotted. Error bars, S.D.; *, p<0.05; **, p<0.01 versus N2; †, p<0.05 versus *daf-41(ok3052);* n.s., no significant difference by t-test. (H) Gene expression of *daf-41* was slightly reduced at 15°C, but no significant differences were measured between 20°C and 25°C. Error bars, S.D. *, p<0.05; n.s., no significance by t-test.

Because *daf-41* mutants showed clear temperature dependent dauer and stress resistance phenotypes, we wondered whether *daf-41* would influence ageing at various temperatures. Increasing temperature is well known to reduce longevity in ectotherms, including wild type *C*. *elegans* (Fig. 3A-C, Table 1). *daf-41* mutants exhibited an altered temperature dependent longevity, revealing a leveling out of the lifespan curves to those typically seen at 20°C: animals were long lived at 25°C, normal lived at 20°C, and short lived at 15°C relative to wild type (Fig. 3A-E). Other PGS mutants showed normal temperature dependent lifespan phenotypes (Fig. 3A-C, Table 1).

**Table 1:** Ageing data of daf-41 mutants.

Genotype	Temperature	Mean of Mean Lifespan				Mean of Median Lifespan				Mean of Maximum Lifespan				% Change in Mean vs N2	% Change in Mean vs *daf-41(ok3052)*	% Change in Mean vs 20°C	Total Worms in 3 Experiments	Total Omitted Worms in 3 Experiments
N2	15°C	30.7 ± 0.8	^¶¶^			30.7 ± 0.7	^¶¶^			48.0 ± 0.0	^¶^					35.8%	369	111
*daf-41(ok3052)*	15°C	21.8 ± 0.8	*			21.3 ± 0.7	**			35.3 ± 2.9	*	^¶^		-28.9%		4.1%	286	194
*daf-41(ok3015)*	15°C	25.0 ± 0.4	*	^†^	^¶^	26.0 ± 0.0	**	^††^	^¶^	39.3 ± 2.4	*	^¶^		-18.6%	14.6%	7.4%	403	77
*daf-2(e1370)*	15°C	46.3 ± 0.5	*	^††^		46.0 ± 1.2	**	^††^		85.3 ± 1.8	**	^††^	^¶^	50.9%	112.3%	11.7%	404	76
*pges-2(ok3316)*	15°C	25.0 ± 1.2	*	^¶^		24.7 ± 1.3	*	^¶^		40.0 ± 2.0	*	^¶^		-18.5%	14.7%	24.1%	397	83
*gst-4&msp-38(ok2358)*	15°C	30.6 ± 1.3	*	^†^		30.7 ± 1.8	^†^			53.3 ± 2.7	^†^	^¶^		-0.3%	40.3%	29.7%	352	128
N2	20°C	22.6 ± 0.4				23.3 ± 0.7				36.7 ± 2.4							288	132
*daf-41(ok3052)*	20°C	20.9 ± 0.4				21.3 ± 0.7				38.7 ± 2.4				-7.2%			267	153
*daf-41(ok3015)*	20°C	23.3 ± 0.0	^†^			23.0 ± 0.6	^†^			38.0 ± 1.2				3.0%	11.0%		290	130
*daf-2(e1370)*	20°C	41.5 ± 4.7	*	^†^		41.3 ± 4.4	*	^†^		83.3 ± 5.9	**	^††^		83.6%	97.9%		256	164
*pges-2(ok3316)*	20°C	20.1 ± 0.3	**			21.3 ± 0.7				33.3 ± 1.3				-10.8%	-3.8%		308	112
*gst-4&msp-38(ok2358)*	20°C	23.6 ± 0.5				24.5 ± 0.4				39.0 ± 0.8				4.4%	12.6%		312	108
N2	25°C	13.6 ± 0.3	^¶¶^			14.0 ± 0.0	^¶¶^			22.3 ± 0.3	^¶^					-39.7%	615	15
*daf-41(ok3052)*	25°C	18.8 ± 1.2	*			19.0 ± 1.0	*			30.3 ± 2.6	*			37.7%		-10.5%	619	11
*daf-41(ok3015)*	25°C	17.1 ± 0.5	**	^¶¶^		17.7 ± 0.9	*	^¶^		29.0 ± 1.0	*	^¶¶^		25.9%	-8.6%	-26.3%	608	22
*daf-2(e1370)*	25°C	16.7 ± 0.6	*	^¶^		16.0 ± 1.2	^¶¶^			31.0 ± 1.0	*	^¶¶^		22.9%	-10.7%	-59.6%	613	17
*pges-2(ok3316)*	25°C	13.4 ± 0.4	^†^	^¶¶^		14.0 ± 0.0	^†^	^¶¶^		22.3 ± 1.2	^†^	^¶^		-1.7%	-28.6%	-33.5%	600	30
*gst-4&msp-38(ok2358)*	25°C	12.9 ± 0.4	*	^†^	^¶¶^	12.7 ± 0.7	^††^	^¶^		21.7 ± 0.3	^†^	^¶¶^		-5.6%	-31.4%	-45.5%	619	11
**Transgenic rescue**																		
N2	15°C	34.2 ± 1.2	^¶^			35.0 ± 1.5				50.3 ± 6.4						66.2%	283	167
*daf-41(ok3052)*	15°C	24.9 ± 0.4	*			24.7 ± 0.7	*			34.3 ± 1.9				-27.0%		3.2%	130	320
*N2;dhEx906*	15°C	25.1 ± 0.3	*			24.7 ± 0.7	*			37.0 ± 0.6				-26.6%	0.6%	28.3%	142	308
*daf-41;dhEx906*	15°C	29.4 ± 1.0	*	^†^	^¶^	28.0 ± 1.2	**	^¶^		42.3 ± 3.0				-13.9%	17.9%	42.1%	142	308
N2	20°C	20.6 ± 2.0				20.0 ± 3.3				34.0 ± 1.6							246	204
*daf-41(ok3052)*	20°C	24.2 ± 0.4				24.0 ± 0.0				38.5 ± 1.2	*			17.5%			308	143
*N2;dhEx906*	20°C	19.6 ± 1.6				19.5 ± 1.2	^††^			31.0 ± 2.4				-4.9%	-19.0%		278	173
*daf-41;dhEx906*	20°C	20.7 ± 0.9				20.5 ± 0.4	^†^			34.0 ± 0.0				0.7%	-14.3%		309	141
N2	25°C	12.7 ± 0.6				12.7 ± 0.7				21.3 ± 1.9						-38.4%	394	91
*daf-41(ok3052)*	25°C	15.3 ± 0.6	**	^¶¶^		15.7 ± 0.7	*	^¶¶^		27.3 ± 0.9	*	^¶^		20.6%		-36.8%	430	55
*N2;dhEx906*	25°C	11.7 ± 0.7	*	^†^	^¶^	13.0 ± 0.8	^††^	^¶^		20.5 ± 1.2	^†^			-7.5%	-23.3%	-40.1%	285	40
*daf-41;dhEx906*	25°C	12.5 ± 0.3	^†^			13.7 ± 0.9	^¶¶^			18.7 ± 0.3	^††^	^¶¶^		-1.7%	-18.5%	-39.8%	474	51

Mean, median and maximum lifespan are shown

*, p<0.05

**, p<0.01 versus N2,

^†^, p<0.05

^††^, p<0.01 versus daf-41(ok3052),

^¶^, p<0.05

^¶¶^, p<0.01 versus 20°C by t-test.

To determine if the observed longevity phenotypes were due to lesions in *daf-41*, we performed rescue experiments with the *daf-41(+)* transgene. As expected, *daf-41(ok3052)* mutant animals harboring *daf-41(+)* were readily rescued for Daf-c phenotypes, while the *pges-2(+)* control transgene expressed under *daf-41* regulatory elements did not rescue (Fig. 3F). We next tested the influence of the transgenes on ageing. First, we found that overexpression of *daf-41* and *pges-2* had no effect on ageing in both N2 and *daf-41(ok3052)* at 20°C (Fig. 3G and S5A Fig). However, the *daf-41* transgene clearly reversed the *daf-41(ok3052)* longevity phenotype at 25°C, and partially rescued the short-lived phenotype at 15°C (Fig. 3G and S5B-C Fig). In sum, these results reveal that *daf-41(+)* regulates longevity in response to temperature. Consistent with a role in thermal regulated processes, *daf-41* mRNA increased modestly with temperature (Fig. 3H).

Because metabolic rates increase with temperature and vary inversely with longevity, we wondered whether *daf-41* mutants altered mitochondrial metabolism. When we measured O_2_ consumption of *daf-41* mutants at different temperatures, however, we saw no significant differences from wild type (S5D Fig). We also examined reproductive potential of *daf-41(ok3052)* worms and found that mutants produced nearly the same number of progeny as wild type at 20°C (S5E Fig).

### *daf-41* regulates longevity via insulin signaling and the heat stress response

**Fig 4:**
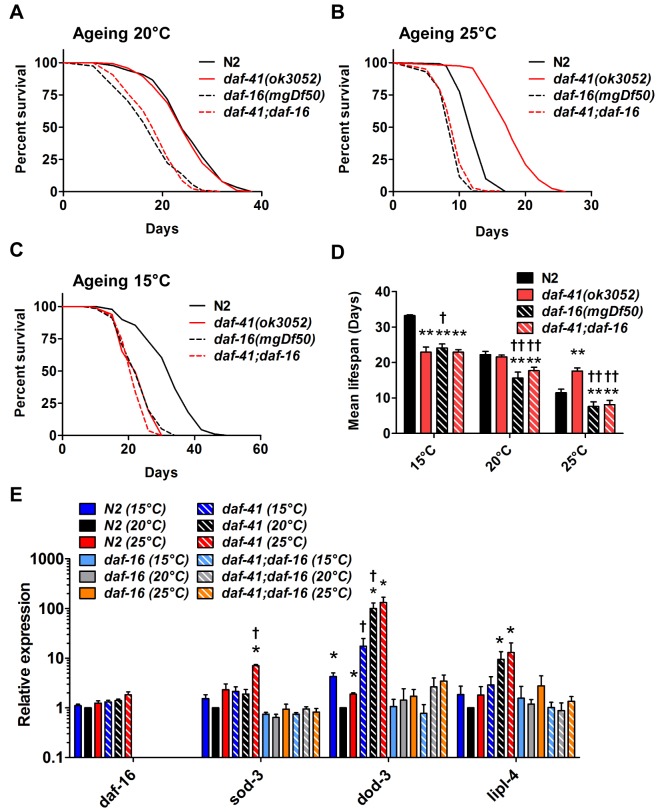
*daf-41* longevity is dependent on *daf-16*/FOXO. (A) *daf-16(mgDf50)* equally reduced the lifespan of *daf-41(ok3052)* and N2 worms at 20°C. (B) *daf-16(mgDf50)* abolished *daf-41* longevity at 25°C. (C) *daf-16(mgDf50)* did not further reduce the short life span of *daf-41(ok3052)* worms at 15°C. (D) Mean lifespan from 3 individual experiments were plotted for the indicated genotypes. Error bars; S.D.; **,p<0.01 versus N2; ††, p<0.01 versus *daf-41(ok3052)* by t-test. (E) DAF-16 target genes, *sod-3*, *dod-3* and *lipl-4*, were significantly upregulated in response to warm temperature in *daf-41(ok3052)* relative to N2. n = 4 biological replicates. Error bars, S.E.M; *, p<0.05 versus N2 of 20°C; †, p<0.05 versus *daf-41(ok3052)* of 20°C by t-test.

To better understand the nature of *daf-41* longevity, we next performed genetic epistasis experiments with known longevity regulators, including *daf-16*/FOXO, *hsf-1*/heat shock factor, *daf-12*/FXR steroid receptor, and *daf-10*/IFT122 [8,51–54]. Intriguingly, *daf-16* was epistatic at all temperatures. At 25°C *daf-16(mgDf50)* completely abolished the longevity of *daf-41(ok3052)* (Fig. 4B, Table 2). At 20°C *daf-16* mutation reduced lifespan of *daf-41(ok3052)* similar to wild type N2 (Fig. 4A). At 15°C, *daf-16* mutation did not further reduce the short lifespan of *daf-41(ok3052)* mutants (Fig. 4C). Moreover, *daf-16* mutation had the effect of restoring temperature dependent regulation of lifespan to *daf-41* mutants (Fig. 4D, Table 2). Although *daf-16* mRNA levels were little affected by *daf-41*, several major target genes of *daf-16*, including *sod-3*, *dod-3*, and *lipl-4* [55] were elevated in *daf-41(ok3052)* in a *daf-16* dependent manner (Fig. 4E). No differences in DAF-16 nuclear localization were seen between WT and *daf-41(ok3052)*, since both showed moderately elevated translocation at 25°C, (S6A-B Fig). These data support the notion that *daf-41(+)*, either directly or indirectly inhibits the activity but not localization of DAF-16/FOXO at elevated temperatures. We therefore performed qRT-PCR analysis for insulin like peptides and found that expression of several *ins* genes were changed by temperature shift and in *daf-41(ok3052)* worms. In particular, *ins-1*, *ins-5*, *ins-7*, *ins-10*, *ins-11*, *ins-12*, *ins-17*, *ins-18*, *ins-27* and *ins-37* were up-regulated in *daf-41(ok3052)* worms at 25°C, and only *ins-13* was down-regulated at 25°C (S7 Fig), consistent with modulation of IIS.

**Table 2:** Ageing data of daf-41(ok3052) in various mutant backgrounds.

Genotype	Temperature	Mean of Mean Lifespan				Mean of Median Lifespan				Mean of Maximum Lifespan				% Change in Mean vs N2	% Change in Mean vs *daf-41 (ok3052)*	% Change in Mean vs 20°C	Total Worms in 3 Experiments	Total Omitted Worms in 3 Experiments
**vs *daf16(mgDf50)***																		
N2	15°C	33.2 ± 0.2	^¶¶^			34.0 ± 0.0	^¶¶^			54.0 ± 2.3	^¶^					44.9%	256	109
*daf-41(ok3052)*	15°C	22.9 ± 0.8	**			23.3 ± 1.3	**			35.3 ± 2.7	*	^¶^		-30.9%		-4.2%	209	216
*daf16(mgDf50)*	15°C	24.0 ± 0.7	**	^†^	^¶¶^	23.3 ± 1.3	**	^¶¶^		36.7 ± 1.3	*	^¶^		-27.6%	4.9%	54.2%	270	125
*daf-41;daf-16*	15°C	22.9 ± 0.4	**	^¶^		22.0 ± 0.0	**	^¶^		32.7 ± 1.3	**	^¶^		-31.0%	-0.1%	29.4%	303	122
N2	20°C	22.9 ± 1.2				23.3 ± 0.7				35.0 ± 2.5							259	216
*daf-41(ok3052)*	20°C	23.9 ± 1.1				23.3 ± 0.7				37.3 ± 1.8				4.5%			297	178
*daf16(mgDf50)*	20°C	15.6 ± 1.0	**	^††^		15.7 ± 0.9	**	^††^		27.7 ± 2.3	*	^†^		-31.9%	-34.9%		287	188
*daf-41;daf-16*	20°C	17.7 ± 0.6	**	^††^		18.3 ± 0.9	**	^††^		28.7 ± 1.7	*	^†^		-22.7%	-26.1%		403	72
N2	25°C	11.5 ± 0.6	^¶¶^			11.0 ± 0.6	^¶¶^			18.3 ± 0.9	^¶^					-50.0%	357	3
*daf-41(ok3052)*	25°C	17.0 ± 0.6	**	^¶¶^		17.3 ± 0.7	**	^¶¶^		28.0 ± 1.2	**	^¶^		48.4%		-29.0%	357	3
*daf16(mgDf50)*	25°C	7.6 ± 0.8	**	^††^	^¶¶^	7.3 ± 1.3	*	^††^	^¶^	13.0 ± 0.6	*	^††^	^¶¶^	-33.7%	-55.3%	-51.3%	415	5
*daf-41;daf-16*	25°C	8.1 ± 0.7	**	^††^	^¶¶^	8.3 ± 1.2	*	^†^	^¶^	14.0 ± 1.5	^†^	^¶¶^		-29.1%	-52.2%	-54.1%	389	31
**vs *hsf-1(sy441)***																		
N2	15°C	35.7 ± 0.2	^¶¶^			37.0 ± 1.0	^¶¶^			52.7 ± 1.3	^¶^					38.2%	348	122
*daf-41(ok3052)*	15°C	26.8 ± 0.2	**	^¶¶^		26.0 ± 0.0	**	^¶¶^		39.7 ± 2.3	**	^¶^		-24.7%		23.1%	276	194
*hsf-1(sy441)*	15°C	26.7 ± 1.3	**	^¶^		27.3 ± 1.3	*	^¶^		39.3 ± 1.3	*	^¶^		-25.1%	-0.5%	61.8%	359	111
*daf-41;hsf-1*	15°C	18.5 ± 0.6	**	^††^	^¶^	18.0 ± 0.0	**	^††^	^¶^	34.3 ± 0.3	**	^¶^		-48.2%	-31.2%	44.9%	398	72
N2	20°C	25.8 ± 0.3				28.0 ± 0.0				41.3 ± 2.4							406	103
*daf-41(ok3052)*	20°C	21.8 ± 0.2	**			22.3 ± 0.3	**			40.0 ± 0.0				-15.5%			367	26
*hsf-1(sy441)*	20°C	16.5 ± 0.3	**	^††^		18.3 ± 0.3	**	^†^		28.7 ± 0.7	*	^††^		-36.0%	-24.3%		444	35
*daf-41;hsf-1*	20°C	12.7 ± 1.0	**	^††^		12.3 ± 1.5	**	^†^		28.0 ± 1.2	*	^††^		-50.6%	-41.5%		435	18
N2	25°C	12.8 ± 0.8	^¶¶^			14.0 ± 1.2	^¶¶^			20.0 ± 1.2	^¶¶^					-50.2%	453	17
*daf-41(ok3052)*	25°C	17.2 ± 0.9	*	^¶^		17.3 ± 0.7	^¶¶^			28.0 ± 1.2	**	^¶¶^		34.0%		-21.0%	453	35
*hsf-1(sy441)*	25°C	9.8 ± 0.6	**	^†^	^¶¶^	9.3 ± 0.7	**	^†^	^¶¶^	16.3 ± 0.9	*	^††^	^¶¶^	-23.9%	-43.2%	-40.8%	435	43
*daf-41;hsf-1*	25°C	9.7 ± 0.6	**	^††^	^¶^	9.3 ± 1.3	^†^	^¶^		18.0 ± 1.2	^††^	^¶^		-24.4%	-43.6%	-23.8%	427	20
**vs *daf-21(p673)***																		
N2	15°C	33.9 ± 0.9	^¶¶^			34.0 ± 0.0	^¶¶^			54.0 ± 4.6	^¶^					46.9%	280	170
*daf-41(ok3052)*	15°C	26.3 ± 0.5	**	^¶^		26.0 ± 0.0	**			43.3 ± 1.3	*			-22.5%		13.9%	219	231
*daf-21(p673)*	15°C	51.8 ± 0.8	**	^††^	^¶¶^	52.7 ± 1.3	**	^††^	^¶^	95.3 ± 8.7	*	^†^	^¶^	52.7%	97.1%	106.5%	257	193
*daf-41;daf-21*	15°C	46.4 ± 1.5	**	^††^	^¶¶^	46.0 ± 2.3	*	^††^	^¶¶^	75.3 ± 2.7	*	^††^	^¶^	36.8%	76.5%	81.5%	297	153
N2	20°C	23.1 ± 0.1				24.7 ± 0.7				36.3 ± 2.3							406	103
*daf-41(ok3052)*	20°C	23.1 ± 0.5				24.0 ± 1.2				42.0 ± 2.0				-0.1%			367	26
*daf-21(p673)*	20°C	25.1 ± 1.4				22.7 ± 3.7				50.7 ± 0.7	*	^†^		8.6%	8.7%		444	35
*daf-41;daf-21*	20°C	25.6 ± 0.3	*	^†^		26.0 ± 0.0				48.7 ± 3.7				10.7%	10.8%		435	18
N2	25°C	12.9 ± 0.2	^¶¶^			14.0 ± 0.0	^¶¶^			20.7 ± 0.7	^¶^					-44.1%	402	48
*daf-41(ok3052)*	25°C	18.8 ± 1.1	*	^¶^		19.3 ± 1.8	*			31.3 ± 1.8	**	^¶¶^		45.6%		-18.5%	353	97
*daf-21(p673)*	25°C	14.5 ± 0.7	^†^	^¶^		14.0 ± 2.0				26.7 ± 0.7	*	^†^	^¶¶^	11.9%	-23.2%	-42.4%	401	49
*daf-41;daf-21*	25°C	13.8 ± 0.5	^†^	^¶¶^		13.3 ± 0.7	^†^	^¶¶^		24.0 ± 1.2	*	^†^	^¶¶^	6.4%	-26.9%	-46.2%	365	85
**vs *daf-12(rh61rh411)***																		
N2	15°C	33.5 ± 0.1				34.5 ± 0.4	^¶^			60.0 ± 1.6	^¶^					46.5%	258	155
*daf-41(ok3052)*	15°C	21.5 ± 0.1	**			22.0 ± 0.0	*			36.5 ± 1.2	*	^¶^		-35.8%		-10.1%	224	234
*daf-12(rh61rh411)*	15°C	26.0 ± 0.8	**	^¶^		26.0 ± 0.0	*	^††^		48.0 ± 4.9	^¶^			-22.4%	20.9%	65.3%	368	128
*daf-41;daf-12*	15°C	27.1 ± 2.5	**			28.5 ± 5.3	**			44.5 ± 2.0	*	^¶^		-19.2%	25.9%	42.6%	345	113
N2	20°C	22.9 ± 1.2				23.3 ± 0.7				35.0 ± 2.5							259	216
*daf-41(ok3052)*	20°C	23.9 ± 1.1				23.3 ± 0.7				37.3 ± 1.8				4.5%			297	178
*daf-12(rh61rh411)*	20°C	15.7 ± 0.4	*	^†^		14.7 ± 1.2	*	^†^		30.3 ± 1.7	*	^††^		-31.3%	-34.2%		267	208
*daf-41;daf-12*	20°C	19.0 ± 0.4	^†^			17.2 ± 0.7	*	^†^		34.7 ± 1.5	^††^			-17.0%	-20.6%		242	233
N2	25°C	10.4 ± 0.6				9.5 ± 1.2				18.0 ± 1.6						-54.8%	402	3
*daf-41(ok3052)*	25°C	16.4 ± 0.2	*			16.0 ± 0.0	^¶¶^			29.0 ± 0.8	*			58.1%		-31.6%	395	11
*daf-12(rh61rh411)*	25°C	7.5 ± 0.3	^††^	^¶^		7.0 ± 0.8	^†^			16.0 ± 0.0	^†^			-27.6%	-54.2%	-52.4%	473	23
*daf-41;daf-12*	25°C	12.4 ± 0.4	*	^¶¶^		12.5 ± 0.4	^†^	^¶^		18.0 ± 1.6	^†^			19.4%	-24.5%	-35.0%	371	35
**vs *daf-10(e1387)***																		
N2	15°C	33.4 ± 0.2	^¶¶^			34.3 ± 0.3	^¶¶^			56.7 ± 3.5	^¶^					45.6%	253	142
*daf-41(ok3052)*	15°C	22.5 ± 1.0	**			23.3 ± 1.3	**			37.0 ± 1.0	*	^¶^		-32.5%		-6.0%	240	215
*daf-10(e1387)*	15°C	42.6 ± 2.3	**	^††^	^¶^	43.7 ± 2.8	*	^††^	^¶^	69.7 ± 4.6	^†^	^¶^		27.7%	89.2%	52.3%	179	331
*daf-41;daf-10*	15°C	35.2 ± 4.1	**	^†^		31.3 ± 4.8				74.0 ± 6.1	*	^†^	^¶^	5.6%	56.4%	26.2%	241	299
N2	20°C	22.9 ± 1.2				23.3 ± 0.7				35.0 ± 2.5							259	216
*daf-41(ok3052)*	20°C	23.9 ± 1.1				23.3 ± 0.7				37.3 ± 1.8				4.5%			297	178
*daf-10(e1387)*	20°C	28.0 ± 1.3	*			29.7 ± 1.5	*	^†^		46.3 ± 2.8	*	^†^		22.1%	16.8%		154	321
*daf-41;daf-10*	20°C	27.9 ± 1.0	**	^†^		27.3 ± 0.3	*	^†^		54.0 ± 3.5	**	^†^		21.8%	16.5%		246	229
N2	25°C	10.5 ± 0.4	^¶¶^			9.7 ± 0.9	^¶¶^			18.0 ± 1.2	^¶^					-54.3%	386	4
*daf-41(ok3052)*	25°C	16.4 ± 0.1	**	^¶¶^		16.7 ± 0.7	**	^¶¶^		28.7 ± 0.7	**	^¶^		57.1%		-31.3%	383	7
*daf-10(e1387)*	25°C	12.1 ± 0.4	^††^	^¶¶^		11.7 ± 0.3	^††^	^¶¶^		21.7 ± 0.3	*	^††^	^¶¶^	15.5%	-26.5%	-56.8%	444	66
*daf-41;daf-10*	25°C	15.5 ± 1.4	*	^¶¶^		15.7 ± 1.5	*	^¶¶^		28.7 ± 1.8	*	^¶¶^		48.3%	-5.6%	-44.4%	363	57
**vs *gcy* triple [*gcy-23(nj37) gcy-8(oy44) gcy-18(nj38)*]**																		
N2	15°C	35.7 ± 0.2	^¶¶^			37.0 ± 1.0	^¶¶^			52.7 ± 1.3	^¶^					38.2%	348	122
*daf-41(ok3052)*	15°C	26.8 ± 0.2	**	^¶¶^		26.0 ± 0.0	**	^¶¶^		39.7 ± 2.3	**	^¶^		-24.7%		23.1%	276	194
*gcy* triple	15°C	25.8 ± 0.5	**	^¶¶^		26.0 ± 0.0	**	^¶¶^		35.7 ± 1.2	**	^¶^		-27.7%	-4.0%	60.9%	378	92
*daf-41;gcy* triple	15°C	25.5 ± 1.0	**	^¶¶^		24.7 ± 1.3	**	^¶¶^		37.0 ± 1.0	**	^¶^		-28.4%	-4.9%	75.6%	412	58
N2	20°C	25.8 ± 0.3				28.0 ± 0.0				41.3 ± 2.4							406	103
*daf-41(ok3052)*	20°C	21.8 ± 0.2	**			22.3 ± 0.3	**			40.0 ± 0.0				-15.5%			367	26
*gcy* triple	20°C	16.0 ± 0.2	**	^††^		16.3 ± 0.9	**	^†^		26.7 # 0.7	*	^††^		-37.9%	-26.5%		452	22
*daf-41;gcy* triple	20°C	14.5 ± 0.3	**	^††^		15.3 ± 0.3	**	^††^		23.3 # 1.3	*	^††^		-43.7%	-33.3%		448	0
N2	25°C	12.8 ± 0.8	^¶¶^			14.0 ± 1.2	^¶¶^			20.0 ± 1.2	^¶¶^					-50.2%	453	17
*daf-41(ok3052)*	25°C	17.2 ± 0.9	*	^¶^		17.3 ± 0.7	^¶¶^			28.0 ± 1.2	^¶¶^			34.0%		-21.0%	453	35
*gcy* triple	25°C	10.5 ± 0.6	*	^††^	^¶¶^	10.7 ± 0.7	^††^	^¶¶^		18.0 # 1.2	^††^	^¶^		-17.9%	-38.8%	-34.2%	450	23
*daf-41;gcy* triple	25°C	15.0 ± 0.4	*	^†^		15.7 ± 0.3				22.7 # 1.8	^†^			17.2%	-12.6%	3.5%	447	0

Mean, median and maximum lifespan are shown

*, p<0.05

**, p<0.01 versus N2

^†^, p<0.05;

^††^, p<0.01 versus daf-41(ok3052)

¶, p<0.05

^¶¶^, p<0.01 versus 20°C by t-test.

**Fig 5:**
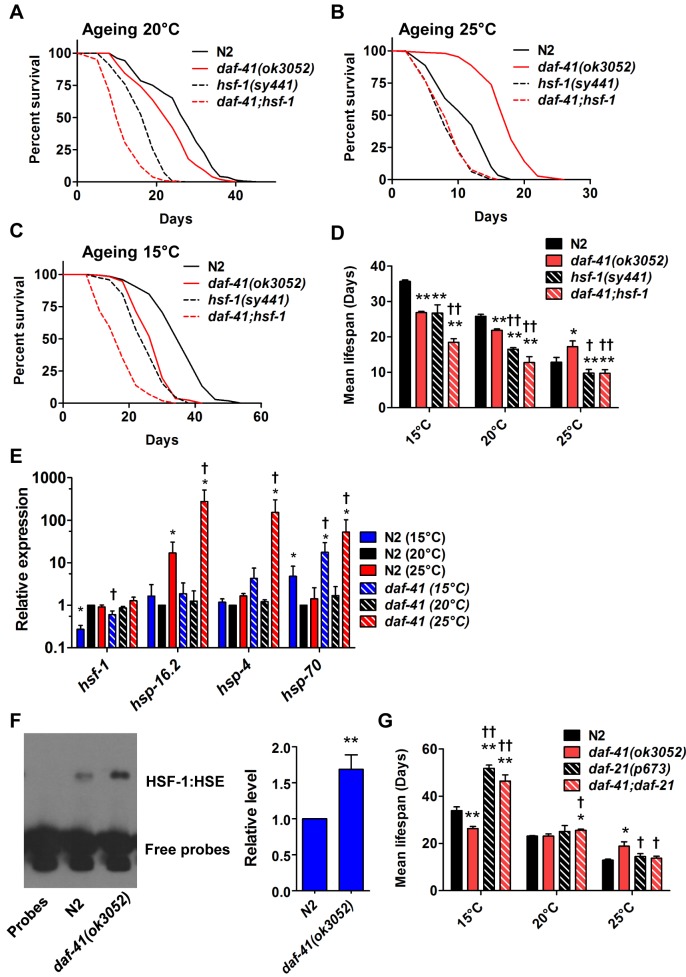
*daf-41(ok3052)* longevity is *hsf-1* dependent. (A) *hsf-1(sy441)* shortened both N2 and *daf-41(ok3052)* life span at 20°C. (B) *hsf-1(sy441)* abolished *daf-41(ok3052)* longevity at 25°C. (C) *hsf-1(sy441)* further reduced *daf-41(ok3052)* short life span at 15°C. (D) Mean lifespan from of 3 individual experiments were plotted for indicated genotypes and conditions. Error bars, S.D.; *, p<0.05; **, p<0.01 versus N2; †, p<0.05; ††, p<0.01 versus *daf-41(ok3052)* by t-test. (E) *daf-41(ok3052)* enhanced the upregulation of HSF-1 target genes, *hsp-16*.*2*, *hsp-4*, and *hsp-70*, in response to warm temperature. n = 4 biological replicates. Error bars, S.E.M; *, p<0.05 versus N2 of 20°C; †, p<0.05 versus *daf-41(ok3052)* of 20°C by t-test. (F) HSF-1 binding activity to HSE was 1.5 fold increased in *daf-41(ok3052)* at 25°C. Error bars, S.E.M; **, p<0.01 versus N2. (G) At 15°C, *daf-21(p673)* mutation enhanced the longevity of N2 and *daf-41(ok3052)*. At 20°C, *daf-21* mutant animals lived slightly longer than N2. At 25°C, *daf-21(p673)* animals lived slightly longer than WT but the mutation reduced longevity in the *daf-41(ok3052)* background.

We next examined genetic interactions with the heat shock transcription factor *hsf-1*, which is required for normal lifespan at various temperatures [8,9]. Longevity of *daf-41(ok3052)* was completely abolished in the *hsf-1(sy441)* background at 25°C, suggesting the two genes work in a unified pathway (Fig. 5B, Table 2). By contrast, the *daf-41;hsf-1* double mutant showed additive short-lived phenotypes at 15°C and 20°C (Fig. 5A, C and D, Table 2) presumably because *hsf-1(sy441)* is non-null. Although *hsf-1* itself was not transcriptionally regulated, major target genes of *hsf-1*, including *hsp-16*.*2*, *hsp-70*, and *hsp-4* [56], showed augmented expression in the *daf-41* background compared to wild type at 25°C (Fig. 5E). These results indicate that *daf-41(+)* directly or indirectly inhibits the activity of HSF-1 at 25°C, to influence transcription and longevity. Recently, it has been reported that HSF-1 forms nuclear foci in response to heat shock but not by reduced IIS [57]. Similarly, we failed to detect foci formation of HSF-1 at 20°C and 25°C *daf-41(ok3052)* mutants (S8A-B Fig).

Given the genetic interactions with *hsf-1* for dauer formation, longevity, and gene transcription described above, we wondered whether *daf-41* affects assembly of HSF-1 nuclear complexes. To measure this, we prepared nuclear extracts from wild type and *daf-41* mutants and performed electrophoretic mobility shift assays on the heat shock factor response element as established previously [58]. First we found that *daf-41* mutation had no effect on mRNA or protein levels of HSF-1 (S8C Fig). Second, we observed that *daf-41* nuclear extracts showed a clear and reproducible 1.5–2 fold higher occupancy of the heat shock factor response element, compared to WT controls (Fig. 5F). These results suggest that DAF-41(+) normally affects the assembly or disassembly of HSF-1 transcriptional complexes.

Because p23 and HSP90 are known to work together, we asked whether they would have similar effects on lifespan. At 25°C, both *daf-41(ok3052)* and *daf-21(p673)gf* worms were longer lived than wild type; however, the *daf-41;daf-21* double mutant had longevity phenotypes more similar to the *daf-21* single mutant (Fig. 5G, S9A-B Fig, Table 2). The convergent behavior at 25°C suggests they could work together at this temperature, similar to dauer. By contrast at 15°C, *daf-21(p673)* worms were extremely long-lived; this longevity was additive to the short-lived phenotype of *daf-41(ok3052)* mutant animals. The distinct behavior of *daf-41/*p23 and *daf-21/hsp90* at 15°C, suggests they may regulate lifespan at this temperature through different pathways (S9C Fig).

**Fig 6:**
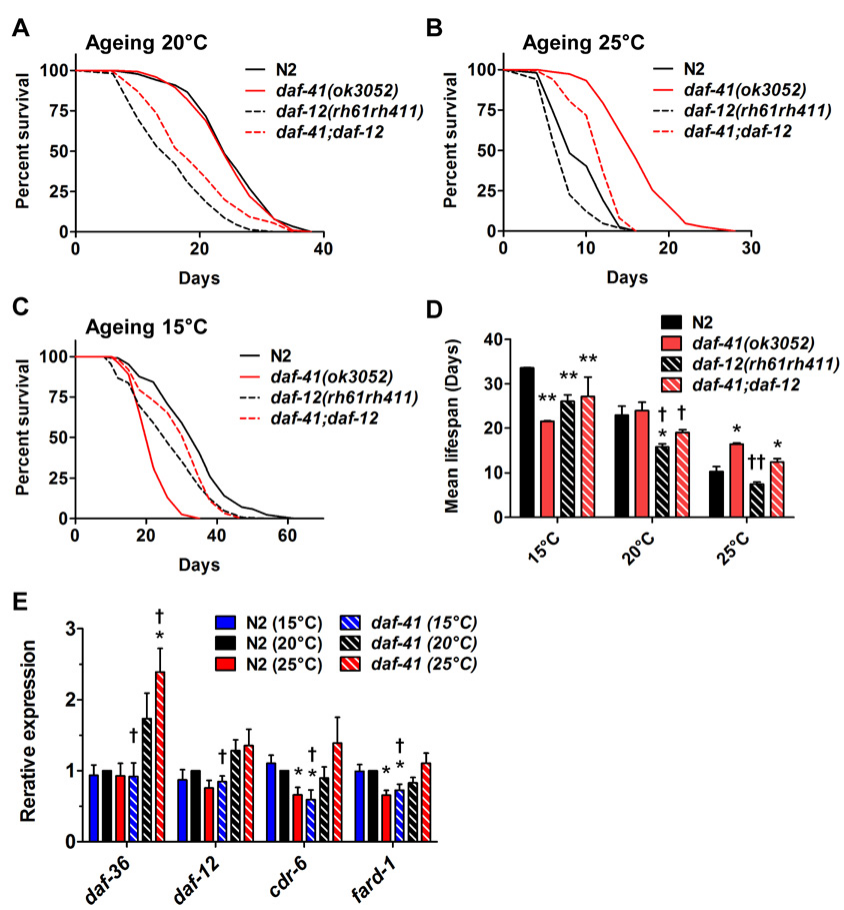
The short life span of *daf-41(ok3052)* at 15°C is *daf-12* dependent. (A-B) *daf-12(rh61rh411)* reduced longevity of *daf-41(ok3052)* at 20°C and 25°C. (C) *daf-12(rh61rh411)* partly rescued the short life span of *daf-41(ok3052)* at 15°C. (D) Mean lifespan from 3 individual experiments are plotted with indicated genotypes and conditions. Error bars, S.D.; *, p<0.05; **, p<0.01 versus N2; †, p<0.05; ††, p<0.01 versus *daf-41(ok3052)* by t-test. (E) Transcriptional targets of DAF-12, *cdr-6* and *fard-1*, were reduced with temperature in N2, but this tendency was reversed in the *daf-41(ok3052)* background. n = 4 biological replicates. Error bars, S.E.M; *, p<0.05 versus N2 of 20°C; †, p<0.05 versus *daf-41(ok3052)* of 20°C by t-test.

The steroid receptor DAF-12 also influenced the longevity phenotypes of *daf-41(ok3052)* (Fig. 6A-D, Table 2). At 25°C, *daf-12* mutation partially reduced the longevity of *daf-41* mutants in an additive manner, but was not epistatic, i.e. *daf-12 daf-41* did not give the same life span as *daf-12* itself. The expression levels of the DA biosynthetic gene, *daf-36*/Rieske, and two DAF-12 target genes, *cdr-6* and *fard-1*, were also increased at higher temperatures. Therefore, *daf-12(+)* could work in parallel, or function as a minor branch of *daf-41* signaling to promote longevity. More interestingly at 15°C, *daf-12* mutation suppressed the short lived phenotypes of *daf-41*, restoring near normal life span. At this temperature, *cdr-6* and *fard-1* genes decreased in expression in *daf-41* mutants relative to WT (Fig. 6E). These interactions suggest that *daf-41(+)* could prevent life shortening properties of DAF-12 steroidal signal transduction at lower temperatures.

**Fig 7:**
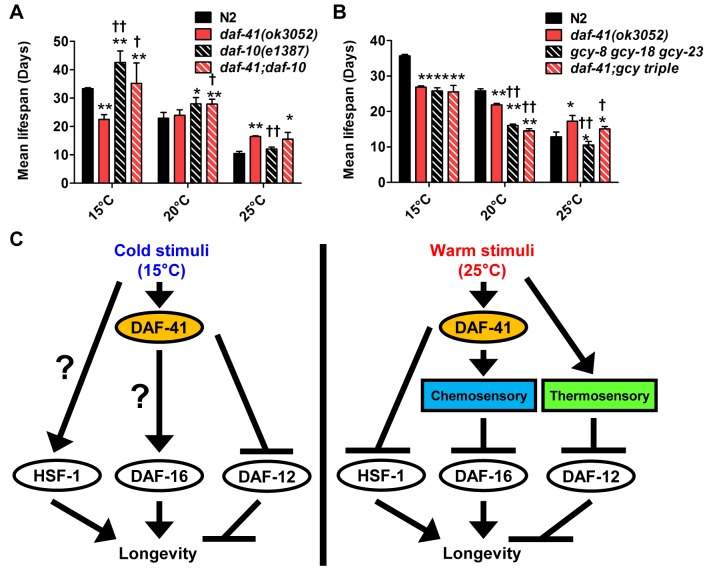
*daf-41* partially interacts with the chemosensory and thermosensory apparatus to regulate longevity. (A) Mean lifespan of 3 individual experiments were plotted. The triple mutant of *gcy-23(nj37) gcy-8(oy44) gcy-18(nj38) (gcy* triple) caused a parallel reduction of lifespan in N2 and *daf-41(ok3052)*, respectively at 25°C. The *gcy* triple mutant did not further shorten the life span of daf*-41(ok3052)* at 15°C. (B) *daf-10* mutation increased lifespan in parallel to *daf-41* at 15°C and 20°C. *daf-10* mutation did not further extend the life span of *daf-41(ok3052)* worms at 25°C. Error bars, S.D.; *, p<0.05; **, p<0.01 versus N2; †, p<0.05; ††, p<0.01 versus *daf-41(ok3052)* by t-test. (C) A schematic model describing the regulatory mechanism of longevity by *daf-41* at different temperatures. At 25°C, *daf-41* negatively regulates the transcriptional activities of DAF-16 and HSF-1 and their down-regulation results in normal life span. Thermotaxis and steroidal signaling may regulate longevity in parallel to *daf-41*. At 15°C, *daf-41* (+) contributes to longevity possibly via *daf-16/*FOXO. *daf-41(+)* may also prevent life shortening activities of *daf-12(+)*, while *hsf-1* may promote longevity in parallel. These are working models that we interpret with caution, and may reflect direct or indirect interactions.

We also examined interactions with *daf-10*/IFT122, which affects neuronal cilia, and is long lived [46,54]. At 15°C and 20°C, *daf-10* mutation did not affect *daf-41* and showed additive phenotypes, i.e. *daf-10* increased lifespan of both N2 and *daf-41(ok3052)* independent of temperature (Fig. 7A, S10A-C Fig, Table 2). At 25°C, however, *daf-41* and *daf-41;daf-10* had similar degrees of lifespan extension, suggesting that the two activities might converge on a common process.

Finally we examined the effect of genes involved in thermotaxis. These genes are implicated in the systemic heat shock response [7]. Moreover, recent work has shown that longevity arising from RNAi knockdown of the *pat-4*/integrin-linked kinase depends completely on genes involved in thermotaxis (*ttx-3* and *gcy-8*), *hsf-1*, but not *daf-16* [59]. For our studies, we used the soluble guanylyl cyclase (*gcy*) triple mutant, *gcy-23(nj37) gcy-8(oy44) gcy-18(nj38)*, which is defective in thermotaxis due to signaling defects in the thermotaxis neurons [60]. As expected, we observed the *gcy* triple mutant to be short-lived at 25°C (Fig. 7B, S10F Fig, Table 2). Unexpectedly, the *gcy* triple mutant equally shortened the lifespan of *daf-41* and wild type, suggesting parallel pathways. At 15°C, the *gcy* triple mutant did not further shorten *daf-41* lifespan, possibly suggesting a convergent mechanism (S10D-E Fig, Table 2).

## Discussion

How temperature influences animal lifespan is not well understood. In this work, we demonstrate that *daf-41*/p23 co-chaperone PTGES3 homolog modulates longevity in response to temperature, and regulates entry and exit from the long-lived dauer stage. On the one hand, *daf-41(+)* promotes normal adult longevity at cold temperature (15°C). On the other hand, *daf-41(+)* limits lifespan at warm temperature (25°C), but has little influence on longevity at 20°C. Thus the overall effect seen in *daf-41* mutants is an equalization of the lifespan curve so that animals appear as if they are living at 20°C. Surprisingly, no difference in respiratory rates (O_2_ consumption) between wild type and *daf-41* animals was detected at these temperatures, suggesting that *daf-41’s* effect on longevity is likely through mechanisms independent of mitochondrial metabolism. These intriguing observations reveal that *daf-41* plays a unifying role in regulatory mechanisms that modulate lifespan in reaction to temperature, and imply that co-chaperone/chaperone complexes may mediate this response.

Although the detailed molecular mechanism by which *daf-41* mediates these temperature dependent effects is not entirely understood, our genetic epistasis experiments suggest that *daf-41* may directly or indirectly impinge on several transcriptional longevity regulatory mechanisms, including DAF-16/FOXO, HSF-1, and DAF-12 steroidal signaling, to regulate lifespan in response to temperature. Several lines of evidence argue that *daf-16*/FOXO is a critical mediator in these circuits. At warm temperature, lifespan extension of *daf-41* mutants was abolished in the *daf-16* mutant background, at low temperatures the *daf-41;daf-16* double mutants did not live shorter than *daf-41* alone, suggesting convergent mechanisms. Overall *daf-16* mutation restored temperature dependent lifespan regulation to *daf-41* mutants. As further support for a link to FOXO function, *daf-16* partly suppressed *daf-41* Daf-c phenotypes, placing *daf-16* downstream for both dauer formation and longevity. Consistent with working in a signaling pathway, we found that *daf-41(+)* negatively regulated transcription of DAF-16 targets (*sod-3*, *dod-3*, *lipl-4*) in response to warm temperature although no clear effect was seen at low temperatures with these genes. Several insulin-like peptides were up or down regulated in *daf-41* mutants in response to warm temperature, suggesting that *daf-41* has the potential to affect IIS. However, we could not detect obvious differences in DAF-16 nuclear localization, as is often seen with mutations that only weakly affect signaling (Henderson et al. 2001). Conceivably *daf-41* could regulate components of IIS or DAF-16 complexes themselves.

Heat shock factor, *hsf-1*, often works in tandem with *daf-16*/FOXO [8,9] and consistently, was also required for *daf-41* induced longevity at elevated temperatures. Accordingly, several *hsf-1* target genes (*hsp-16*.*2*, *hsp-70*, *hsp-4*) were expressed in *daf-41* mutants under these conditions. Although HSF-1 mRNA or protein levels were unchanged in the *daf-41* background, nuclear extracts from *daf-41* mutants showed increased occupancy of the heat shock response element. Importantly, this result suggests that *daf-41(+)* influences either directly or indirectly the assembly/disassembly of the HSF-1 complex. This finding is consistent with observation the p23-HSP90 complex has been shown to inhibit the activity of the HSF-1 complex in other systems [23,25]. The observed short-lived phenotypes of *daf-41* with *hsf-1* at low temperatures were additive, possibly because the *hsf-1* allele is temperature sensitive and non-null [56].

*daf-41*’s interactions with nuclear receptor *daf-12* suggest an intriguing role for steroid signaling. At 15°C, *daf-12* mutation suppressed *daf-41* short-lived phenotypes, suggesting that *daf-41* modulates life extending effects of *daf-12*. Steroid signaling has previously been implicated in lifespan regulation albeit at high temperatures: *daf-9* hypomorphs as well as thermotaxis mutants are particularly short-lived at 25°C, in a manner dependent upon *daf-12* [4]. At these temperatures, thermotaxis loci as well as *hsf-1(+)* promote expression of the hormone biosynthetic gene *daf-9*/CYP27A1 [4,50]. These observations suggest that normal lifespan at 25°C depends on adequate stimulation of steroidal signaling, thereby preventing life shortening activities of the unliganded DAF-12. At 25°C, *daf-12* mutation shortened the life span of *daf-41* to a similar extent as wild type, suggesting parallel pathways. Alternately *daf-12* could contribute partially toward *daf-41* longevity, since *daf-12* target genes as well as *daf-36* increase expression at this temperature. At this point, it is unclear whether the activating or repressing functions of the receptor are responsible for longevity.

In mammals, p23 together with HSP90, immunophilins and other chaperones, binds to unliganded steroid receptors in the cytosol, maintaining the receptor in a primed state competent for rapid ligand binding; upon binding hormone the steroid receptor enters the nucleus where interacts with transcriptional coregulators. Thereafter, p23 helps disassemble transient transcriptional complexes to facilitate hormone sampling and repeated rounds of transcription [15,20–22,26]. The p23-HSP90 complex also regulates type II nuclear receptors, such as the thyroid receptor [20], which constitutively reside in the nucleus, a situation more resembling that of DAF-12. Further studies on the role of steroidal signaling at low and high temperature may help clarify the whether similar mechanisms are at work in *C*. *elegans*.

Interactions with chemosensory mutants suggest a role within the sensory apparatus. Indeed, *daf-41* longevity at warm temperatures converged with *daf-10*/IFT122, and both mutants stimulate *daf-16*, suggesting they could work through a similar chemosensory mechanism [54]. Consistent with a proximal role in chemosensory signaling, *daf-41* mutants exhibited chemosensory deficits and chemosensory mutants suppressed the *daf-41* Daf-c phenotypes. Accordingly, *daf-41* was expressed in several chemosensory neurons including ASE, AWC, ADL, and ASI but was not obviously expressed in the main thermosensory neurons AFD and AIY, although AWC and ASI also reportedly contribute to thermosensation [61]. Ultimately, tissue specific dissection of *daf-41* activities may help illuminate what cells mediate these interactions. It is also noteworthy that *daf-41* did not further shorten the lifespan of the *gcy* triple mutant at 15°C, suggesting it could work through thermotaxis circuits at low temperature.

p23 is known to interact directly with HSP90 to modulate various client proteins [15,16], and recently, *C*. *elegans* p23 and HSP90 have been shown to physically interact in vitro [47]. Consistent with the possibility of working together, both *daf-41/*p23 and *daf-21/*HSP90 act at the level of chemosensory processing with respect to dauer formation (this work; [49]), and modulated one anothers’ dauer and longevity phenotypes at 25°C. That *daf-41* null and *daf-21* gain of function have similar phenotypes could indicate that the wild type activities work in opposition for these processes. On the other hand, *daf-41* and *daf-21* had several divergent phenotypes: whereas *daf-41* Daf-c phenotypes were enhanced by *hsf-1* mutation (Fig. 2H, this work), *daf-21* Daf-c phenotypes were suppressed [50]. Furthermore, whereas *daf-41* mutants were short-lived at 15°C, *daf-21* mutants were long-lived, and regulated life span differently. Thus some p23 phenotypes might arise from HSP90 dependent as well as independent processes, as has been noted previously [26,27]. We interpret these experiments with caution since *daf-21* mutations are gain-of-function and non-null. Intriguingly, HSP90 in *Candida albicans* has been shown to regulate the switch to pseudohyphal growth in response to temperature [62], perhaps analogous to the thermal regulation of dauer formation or longevity.

Based on the observed genetic interactions we suggest the following model for longevity regulation. At elevated temperatures, p23 directly or indirectly inhibits the transcriptional activities of HSF-1 and perhaps DAF-16, thus limiting lifespan at these temperatures, while DAF-12/FXR and thermotaxis signaling work in parallel (Fig. 7C). Given the convergence with *daf-10* longevity, we suggest that *daf-41(+)* might work through the chemosensory apparatus to impinge upon DAF-16. More speculatively, at lower temperatures, p23 stimulates DAF-16/FOXO and inhibits DAF-12/FXR, possibly through the thermotaxis apparatus, while HSF-1 works in parallel to promote lifespan extension.

Given the intriguing genetic interactions described here, it would be interesting to investigate whether DAF-41 and/or HSP90 binds and regulates the activities of HSF-1, DAF-16, or DAF-12 by protein-protein interaction. Alternately DAF-41 could interact with upstream components of these signaling pathways, including kinases, temperature sensitive channels, guanylyl cyclases, or cilia proteins. Conceivably, thermal regulation in these circuits could result from thermal influences on protein-protein interactions.

The results here and elsewhere reveal that regulation of longevity at different temperatures works by distinct mechanisms. This is perhaps not surprising, given that different stresses challenge the organism at low and high temperatures. Moreover, ectotherms must have evolved optima for growth and reproduction within a temperature range. With this in mind, we suggest that *daf-41* could play distinct roles at low and high temperatures. Alternately *daf-41* may be part of an adaptive response to temperature in which optima shifted towards higher temperatures have a consequent tradeoff at lower temperatures, and vice versa. Further elucidation of *daf-41/*p23 complexes and physiology in *C*. *elegans* should help illuminate the mechanism of thermal regulation of metazoan longevity.

## Methods

### Strains

Strains were obtained from the Caenorhabditis Genetics Center (CGC, USA) and National Bio Resource Project (NBRP, Japan). All strains were outcrossed at least 4 times to wild type N2 before further analysis. All strains in this work are itemized in S1 Table. Genotypes of mutants were confirmed by PCR, sequencing and phenotyping. Primer sets for genotyping are listed in S2 Table.

### Ageing experiments

All lifespans were measured as previously described [52]. Strains for ageing experiments were maintained at the respective cultivation temperature of 15°C and 20°C for more than 3 generations before analysis, unless indicated otherwise. Progeny were collected by egg laying and bleaching. Ageing experiments were performed with and without 2′fluoro-5′deoxyuridine (FUdR, Sigma) to prevent contamination of next generation progeny and to reduce bagging phenotypes of Egl mutants. Strains were treated with FUdR as described previously [63]. For ageing experiments at warm temperature, worms were cultivated at 20°C until the young adult stage, then moved to 25°C to start the ageing analysis in order to bypass dauer formation and minimize internal hatching phenotypes. Each experiment started with more than 150 worms. Sterile, escaped, internally hatched, and exploded worms were censored on the day of loss. Experiments were performed at least 3 times and the mean lifespan calculated. Worms were transferred onto new OP plates every 2–3 days from the end of reproductive period, and scored for survival every 2–4 days. Statistical analyses were performed by Kaplan–Meier method with GraphPad Prism software (GraphPad Software, Inc.)

### Generation of transgenic strains

The regions of the *daf-41/ZC395*.*10* promoter, coding region and 3' UTR, as well as *pges-2* coding region and its 3' UTR were amplified by PCR. The promoter region was inserted in front of *gfp* in the L3781 plasmid and coding regions inserted after *gfp*. *daf-41*p::*gfp*::*daf-41*::3'UTR and *daf-41*p::*gfp*::*pges-2*::3'UTR plasmids were confirmed by sequencing and co-injected into *daf-41(ok3052)* worms with *coel*::*RFP* plasmid as an injection marker. Both transgenic strains were outcrossed with N2 to generate the following strains: N2; *daf-41*p::*gfp*::*daf-41*::3'UTR, N2;*daf-41*p::*gfp*::*pges-2*::3'UTR, *daf-41(ok3052)*; *daf-41*p::*gfp*::*daf-41*::3'UTR, and *daf-41(ok3052);daf-41*p:: *gfp*::*pges-2*::3'UTR.

### Dauer formation assays

For dauer formation assays, all strains were maintained at 20°C and eggs collected by egg laying or bleaching. Greater than 50 eggs were transferred onto 3cm OP plates and cultured at 20°C, 22.5°C, 25°C and 27°C, respectively. Dauer formation fraction was typically scored at 60 hrs, and dauer exit ratio was scored at 84 hrs.

### Stress resistance analysis

All strains were maintained at 20°C and Day 1 young adults were used for analysis. For heat stress analysis, 50–100 adult worms were transferred onto fresh 6 cm OP plates and shifted to 35°C. Fraction survival was scored 8 hrs after heat shock. For oxidative stress analysis, 50–100 adult worms were collected by washing off plates and transferred into 24 well plastic plates filled with 20mM of hydrogen peroxide (SIGMA) in M9 buffer. Experiments were performed with 5 biological replicates with 3 technical replicates for each mutant.

### qRT-PCR analysis

Synchronized worms were prepared at different temperatures. Worms were collected in TRIzol (Invitrogen) at L4 stage and frozen in liquid nitrogen. Total RNA was extracted by RNeasy Mini kit (QIAGEN) and Superscript III First Strand Synthesis System (Invitrogen) was used for cDNA generation. qRT-PCR was performed with Power SYBR Green master mix (Applied Biosystems) on a 7900HT Fast Real-Time PCR System (Applied Biosystems). *ama-1* was used as internal control for mRNA quontification. For each analysis, qRT-PCR was performed with at least three biological replicates. Primer sequences are listed in S2 Table.

### Preparation of nuclear protein extracts

WT and *daf-41*(ok3052) worms were harvested and frozen down at day 1 of adulthood. Frozen worm pellets were first homogenized in an equal volume of 2X NPB buffer (20 mM HEPES, pH 7.6, 20 mM KCl, 3 mM MgCl_2_, 2mM EDTA, 0.5 M sucrose, 1 mM dithiothreitol, protease inhibitors, and phosphatase inhibitors) using a Kontes Pellet Pestle tissue grinder. The suspension was then centrifuged (4000 g, 5 min, 4°C) and the pellets were further homogenized by 20 strokes with pestle A of a Dounce homogenizer. The pellet was resuspended in NPB buffer with 0.25% NP-40 and 0.1% Triton-X100, centrifuged again, and washed three more times with the same buffer. The nuclear pellet was extracted with four volumes of HEG buffer (20 mM HEPES, pH 7.9, 0.5 mM EDTA, 10% glycerol, 0.42 M NaCl, 1.5 mM MgCl_2_, and protease inhibitors) at 4°C for 45 min. Finally, the nuclear fraction was collected by centrifugation at 14,000 g for 15 min at 4°C. Protein concentrations were determined with a Bradford assay kit (Bio-Rad, Hercules, CA).

### Electrophoretic mobility shift assays (EMSA)

EMSAs were carried out as previously described [58]. In brief, 1 μg of nuclear extract (described above) was mixed with 1 mg/mL of poly (dI-dC) and 1 nM of a biotin-labeled oligonucleotide containing the heat-shock element (HSE) sequence of *hsp-16*.*1* [58], and incubated for 15 min at room temperature in binding buffer [20 mM HEPES, pH 7.6, 5 mM EDTA, 1 mM dithiothreitol, 150 mM KCl, 50 mM (NH_4_)_2_SO_4_, and 1% Tween 20 (v/v)]. After incubation, the samples were separated by native 3.5% PAGE and the HSF-1::HSE DNA complexes were visualized using a LightShift Chemiluminescent EMSA kit (Pierce, Rockford, IL).

Additional details for DiI staining, chemotaxis analysis, oxygen consumption measurement and microscopy analysis are described in S1 Text.

## Supporting Information

S1 Fig *daf-41p*::*gfp* expression pattern. *dpy-5(e907); sEx10796* [*rCes daf-41p*::*gfp* + *pCeh361*] worms were subjected to fluorescence microscopy and photos taken at different stages (A) focusing on various tissues (B). *daf-41p*::*gfp* was expressed in pharynx, body wall muscles, intestine, many neurons, germ cells and vulva. Scalebar = 0.1mm.
(TIF)

S2 Fig *daf-41* mutants have chemotaxis defects. (A) *daf-41(ok3052)* worms were less attracted by isoamyl alcohol, benzaldehyde, and 2,4,5- trimethylthiazoline compared to WT. Error bars, S.D. *, p<0.05; **, p<0.01 versus N2 by t-test. (B) Neurons of N2 and *daf-41(ok3052)* worms filled with DiI, but not those of *daf-10(e1387)*.
(TIF)

S3 Fig The thermosensory system is not involved in *daf-41* dauer formation. (A-B) Mutations in thermotaxis genes had little effect on *daf-41(ok3052)* dauer formation at 25°C and 27°C. Error bars, S.D. *, p<0.05; **, p<0.01 versus *daf-41(ok3052)* by t-test.
(TIF)

S4 Fig *daf-41* mutation enhances *hsf-1(sy441)* Egl phenotypes. *hsf-1(sy441)* worms showed a weak Egl phenotype that was greatly enhanced in *daf-41(ok3052)*. Error bars, S.D. **, p<0.01 versus N2; ††, p<0.01 versus *daf-41(ok3052)* by t-test.
(TIF)

S5 Fig *daf-41* has no effect on oxygen consumption and fertility. (A-C) *pges-2(+)* transgenes didn’t change lifespan of the *daf-41(ok3052)* worms at any temperatures. *dhEx910* is *pges-2(+)* transgenes under control of *daf-41* 5’ and 3’ regulatory elements. (D) Oxygen consumption of N2 and *daf-41(ok3052)* worms was measured at 15°C, 20°C and 25°C. No significant differences were observed between N2 and *daf-41(ok3052*) mutants. (E) Progeny number of N2 and *daf-41(ok3052*) mutants were measured at 20°C, but no significant differences were seen. It performed with 10 worms. n = 3 biological replicates.
(TIF)

S6 Fig *daf-41* has no effect on nuclear localization of DAF-16. DAF-16::GFP (*muIs109*) was moderately translocated into the nucleus at 25°C in both WT and *daf-41* mutants. RNAi of *daf-2* induced robust nuclear localization of DAF-16. (A) Cyt, Cyt + Nuc, and Nuc indicate mostly cytosolic (Cyt) mostly nuclear localization (Nuc), or both (Cyt + Nuc). n = 4 biological replicates. Error bars, S.E.M; n.s., no significant difference by t-test. (B) Arrows point nuclei. Luc, Luciferase.
(TIF)

S7 Fig *daf-41* and temperature shift affect gene expression of insulin like peptides. qPCR revealed that *ins-1*, *ins-5*, *ins-7*, *ins-10*, *ins-11*, *ins-12*, *ins-17*, *ins-18*, *ins-27* and *ins-37* were upregulated by both temperature shift to 25°C and mutation of *daf-41*. Only *ins-13* was suppressed at 25°C in WT and *daf-41(ok3052)* worms. n = 4 biological replicates. Error bars, S.E.M; *, p<0.05 versus N2 of 20°C by t-test.
(TIF)

S8 Fig *daf-41* has no effect on foci formation of HSF-1 at 25°C. HSF-1 formed foci in the nucleus when induced with heat shock at 37°C for 2min. No such foci were seen in WT and *daf-41(ok3052)* mutants at 25°C (A) n = 4 biological replicates. Error bars, S.E.M; n.s., no significant difference by t-test. (B) Arrows point to nuclei. (C) mRNA and protein levels of HSF-1 were not changed at 25°C in *daf-41(ok3052)* mutants. Error bars, S.D.; n.s., no significant difference by t-test.
(TIF)

S9 Fig Interaction of *daf-41* and *daf-21* for life span phenotypes. (A) At 20°C, *daf-21(p673)* and *daf-41;daf-21* strains lived slightly longer than N2 (B) At 25°C, *daf-21(p673)* worms lived slightly longer than N2 but reduced the longevity of *daf-41(ok3052)*. (C) At 15°C, *daf-21(p673) and* daf*-41;daf-21* strains showed extended longevity relative to N2 and *daf-41(ok3052)* backgrounds.
(TIF)

S10 Fig Interaction of *daf-41* with chemosensory and thermosensory mutants for life span phenotypes. (A-C) *daf-10(e1387)* worms lived longer than N2 at 15°C, 20°C and 25°C. (A-B) *daf-41* regulated lifespan parallel to *daf-10* at 15°C and 20°C, (C) *daf-10* mutation did not further extend longevity in the *daf-41(ok3052)* background at 25°C. (D-F) *gcy* triple mutants [*gcy-8(oy44) gcy-18(nj38) gcy-23(nj37)*] lived shorter than N2 at 15°C, 20°C and 25°C, but (D) the *gcy* triple mutant did not further reduce lifespan in the *daf-41(ok3052)* background at 15°C. (E-F) *daf-41* regulated lifespan parallel to the *gcy* triple mutant at 20°C and 25°C.
(TIF)

S1 Table Strain list. (XLSX)

S2 Table Primer list. (XLSX)

S1 Text Supplemental materials and methods. Additional details for DiI staining, chemotaxis analysis, oxygen consumption measurement and microscopy analysis are described in S1 Text.
(DOCX)
