# Supplementary figures and images for: Co-chaperone p23 Regulates C. elegans Lifespan in Response to Temperature
Source: PLoS Genet. 2015 Apr 1;11(4):e1005023. doi: 10.1371/journal.pgen.1005023 (PMC4382338; doi:10.1371/journal.pgen.1005023)

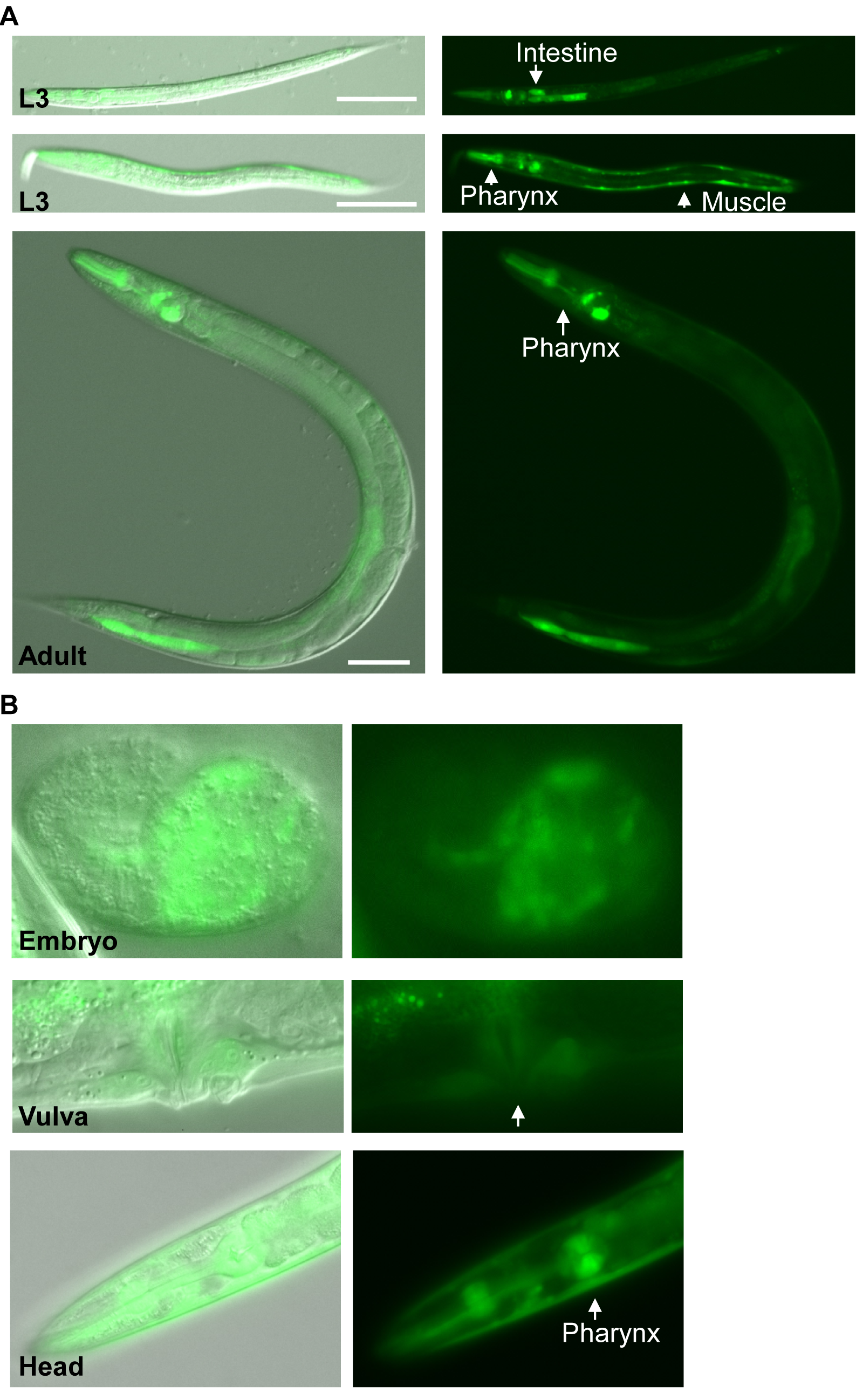

Supplement: S1 Fig — dpy-5(e907); sEx10796 [rCes daf-41p::gfp + pCeh361] worms were subjected to fluorescence microscopy and photos taken at different stages (A) focusing on various tissues (B). daf-41p::gfp was expressed in pharynx, body wall muscles, intestine, many neurons, germ cells and vulva. Scalebar = 0.1mm. (TIF) [file pgen.1005023.s001.tif]

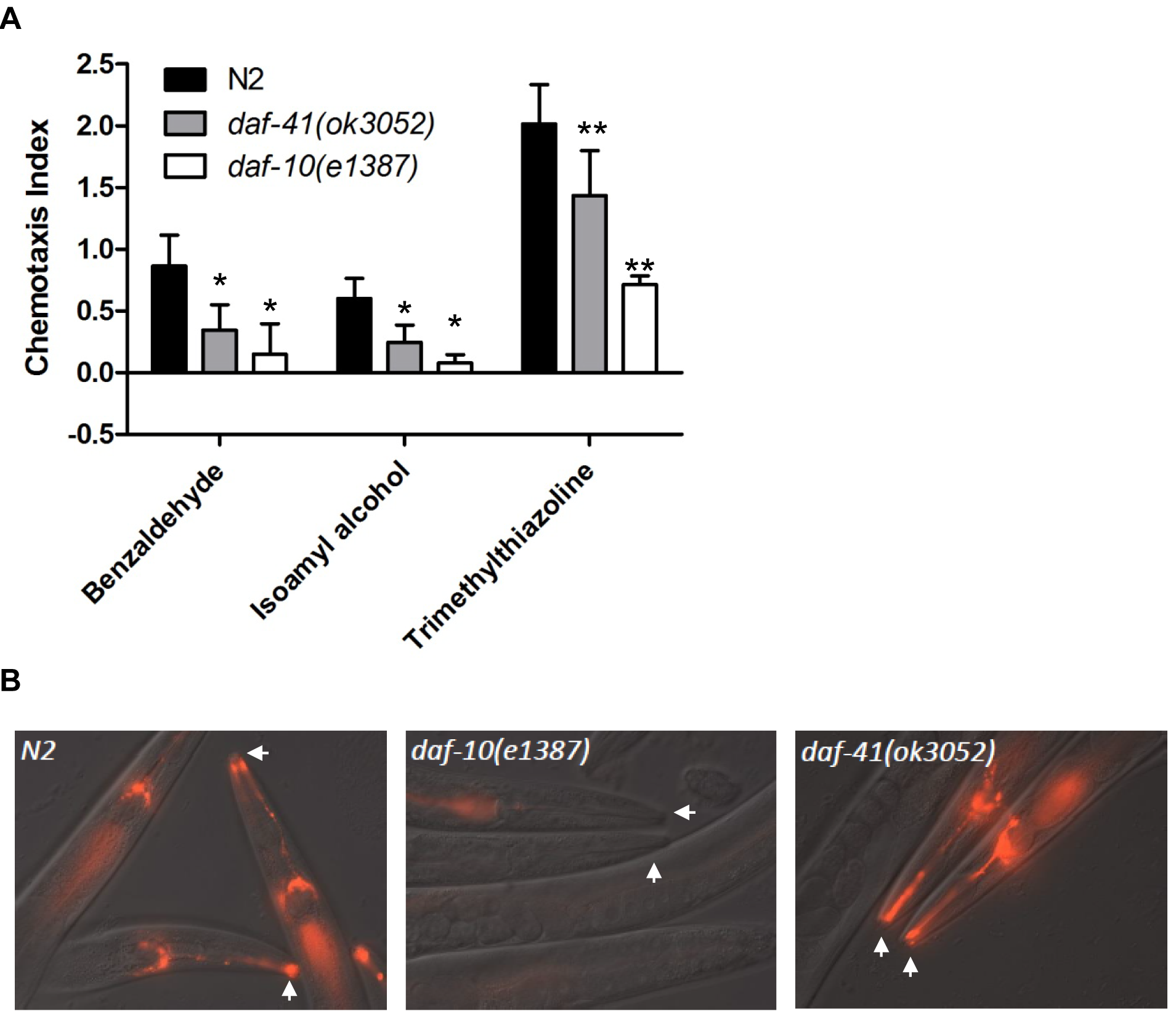

Supplement: S2 Fig — (A) daf-41(ok3052) worms were less attracted by isoamyl alcohol, benzaldehyde, and 2,4,5- trimethylthiazoline compared to WT. Error bars, S.D. *, p<0.05; **, p<0.01 versus N2 by t-test. (B) Neurons of N2 and daf-41(ok3052) worms filled with DiI, but not those of daf-10(e1387). (TIF) [file pgen.1005023.s002.tif]

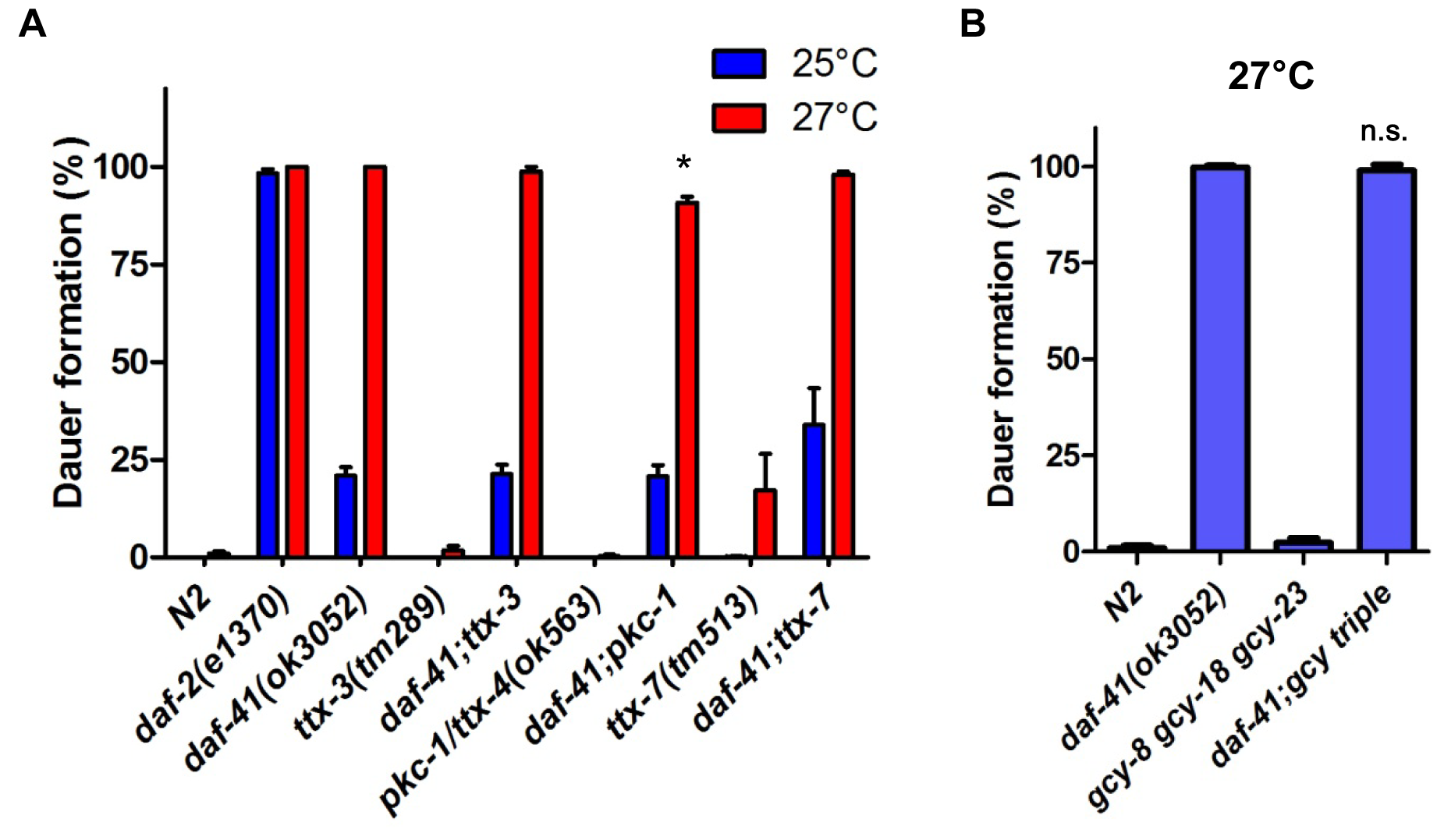

Supplement: S3 Fig — (A-B) Mutations in thermotaxis genes had little effect on daf-41(ok3052) dauer formation at 25°C and 27°C. Error bars, S.D. *, p<0.05; **, p<0.01 versus daf-41(ok3052) by t-test. (TIF) [file pgen.1005023.s003.tif]

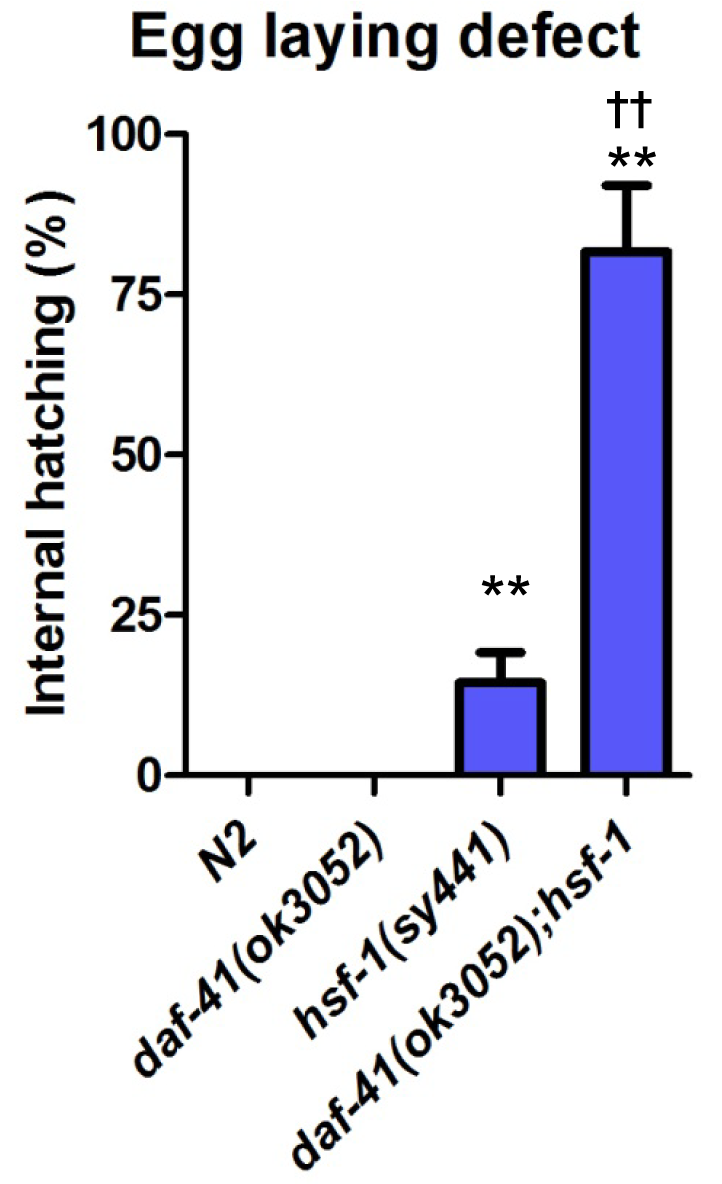

Supplement: S4 Fig — hsf-1(sy441) worms showed a weak Egl phenotype that was greatly enhanced in daf-41(ok3052). Error bars, S.D. **, p<0.01 versus N2; ††, p<0.01 versus daf-41(ok3052) by t-test. (TIF) [file pgen.1005023.s004.tif]

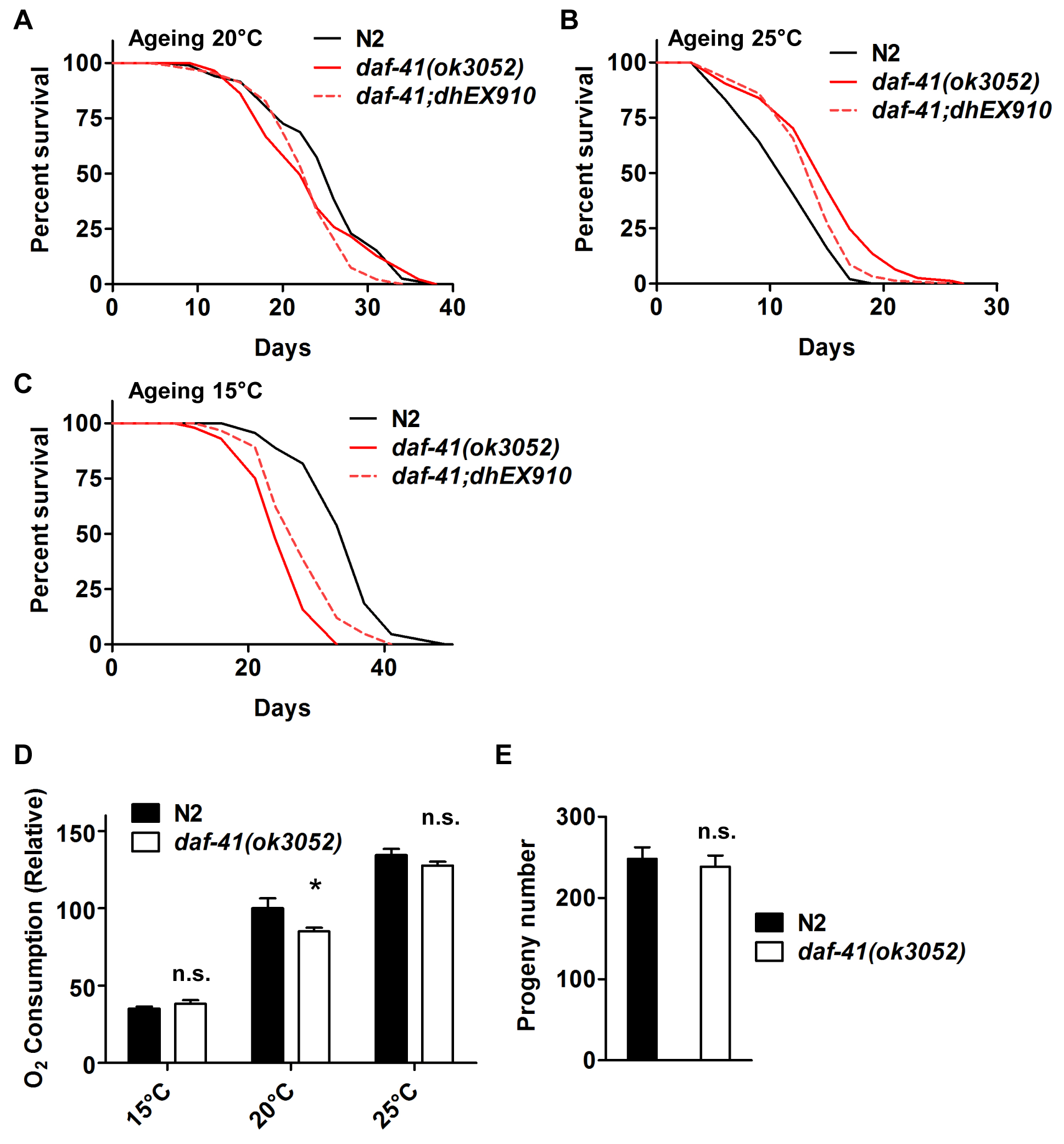

Supplement: S5 Fig — (A-C) pges-2(+) transgenes didn’t change lifespan of the daf-41(ok3052) worms at any temperatures. dhEx910 is pges-2(+) transgenes under control of daf-41 5’ and 3’ regulatory elements. (D) Oxygen consumption of N2 and daf-41(ok3052) worms was measured at 15°C, 20°C and 25°C. No significant differences were observed between N2 and daf-41(ok3052) mutants. (E) Progeny number of N2 and daf-41(ok3052) mutants were measured at 20°C, but no significant differences were seen. It performed with 10 worms. n = 3 biological replicates. (TIF) [file pgen.1005023.s005.tif]

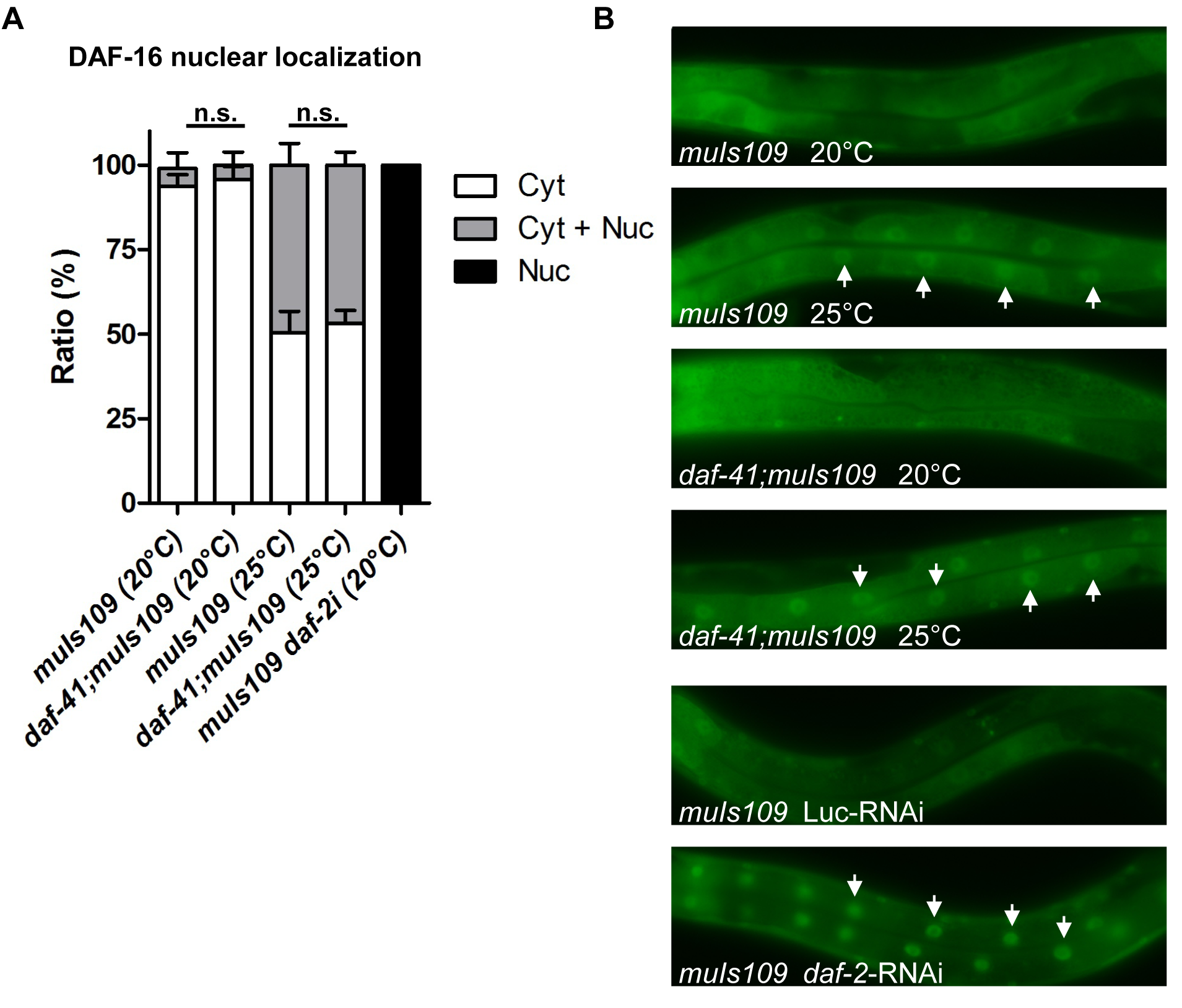

Supplement: S6 Fig — DAF-16::GFP (muIs109) was moderately translocated into the nucleus at 25°C in both WT and daf-41 mutants. RNAi of daf-2 induced robust nuclear localization of DAF-16. (A) Cyt, Cyt + Nuc, and Nuc indicate mostly cytosolic (Cyt) mostly nuclear localization (Nuc), or both (Cyt + Nuc). n = 4 biological replicates. Error bars, S.E.M; n.s., no significant difference by t-test. (B) Arrows point nuclei. Luc, Luciferase. (TIF) [file pgen.1005023.s006.tif]

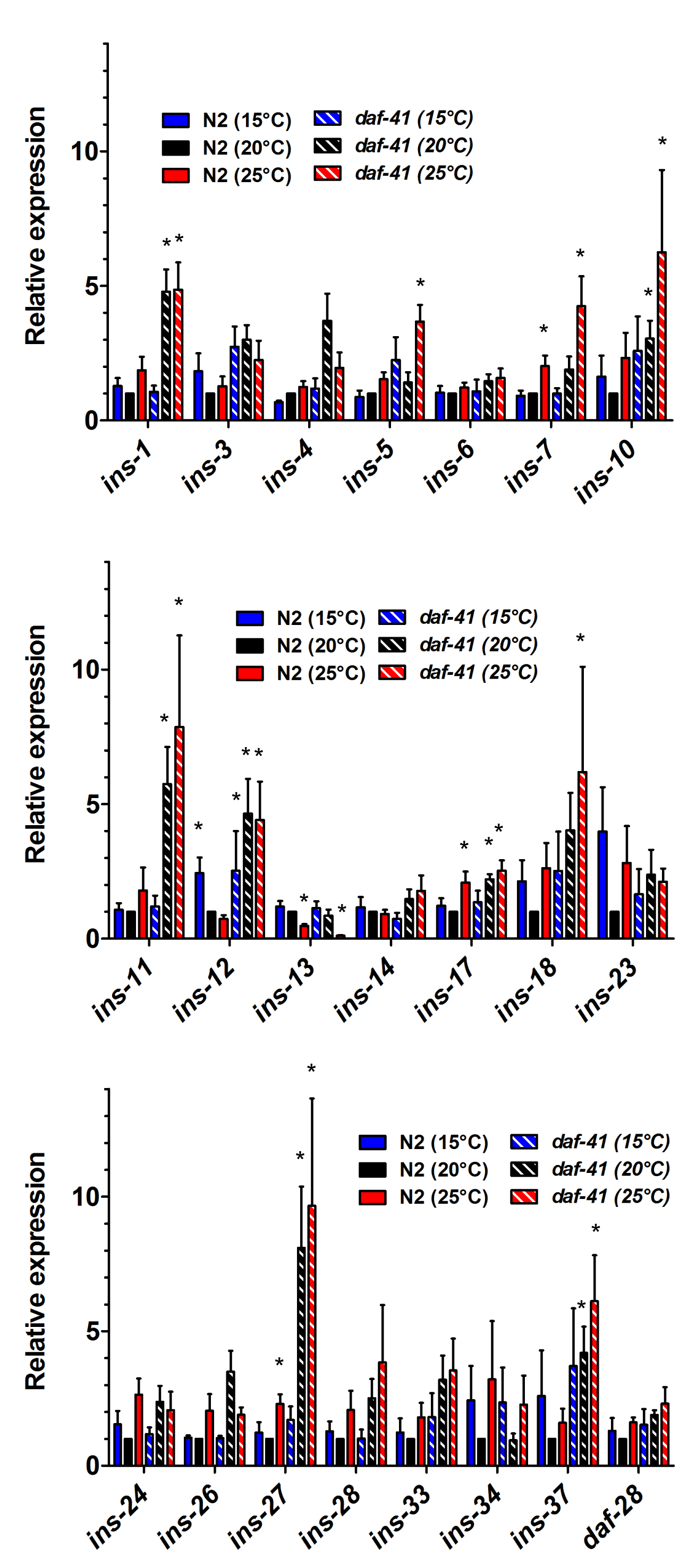

Supplement: S7 Fig — qPCR revealed that ins-1, ins-5, ins-7, ins-10, ins-11, ins-12, ins-17, ins-18, ins-27 and ins-37 were upregulated by both temperature shift to 25°C and mutation of daf-41. Only ins-13 was suppressed at 25°C in WT and daf-41(ok3052) worms. n = 4 biological replicates. Error bars, S.E.M; *, p<0.05 versus N2 of 20°C by t-test. (TIF) [file pgen.1005023.s007.tif]

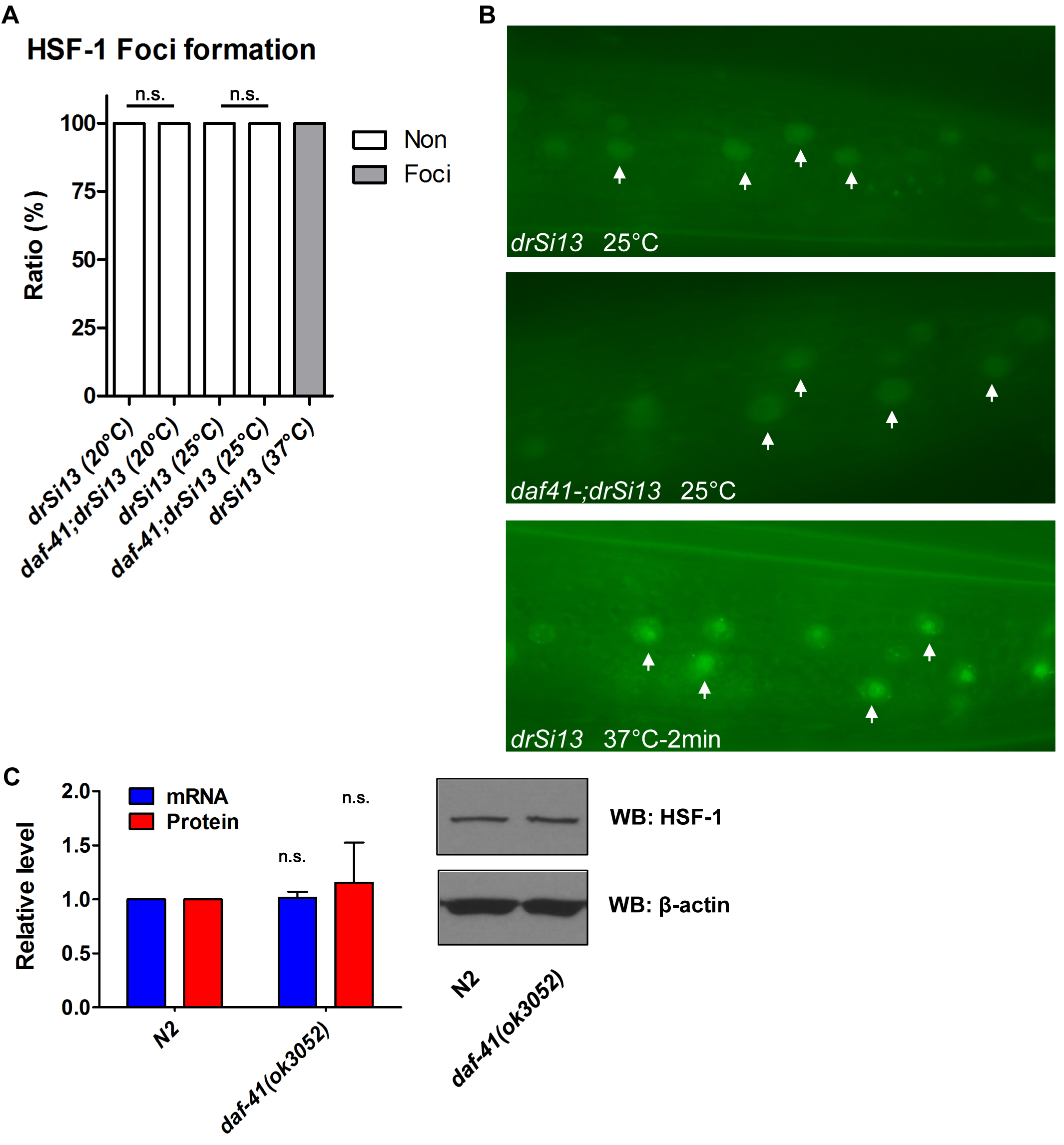

Supplement: S8 Fig — HSF-1 formed foci in the nucleus when induced with heat shock at 37°C for 2min. No such foci were seen in WT and daf-41(ok3052) mutants at 25°C (A) n = 4 biological replicates. Error bars, S.E.M; n.s., no significant difference by t-test. (B) Arrows point to nuclei. (C) mRNA and protein levels of HSF-1 were not changed at 25°C in daf-41(ok3052) mutants. Error bars, S.D.; n.s., no significant difference by t-test. (TIF) [file pgen.1005023.s008.tif]

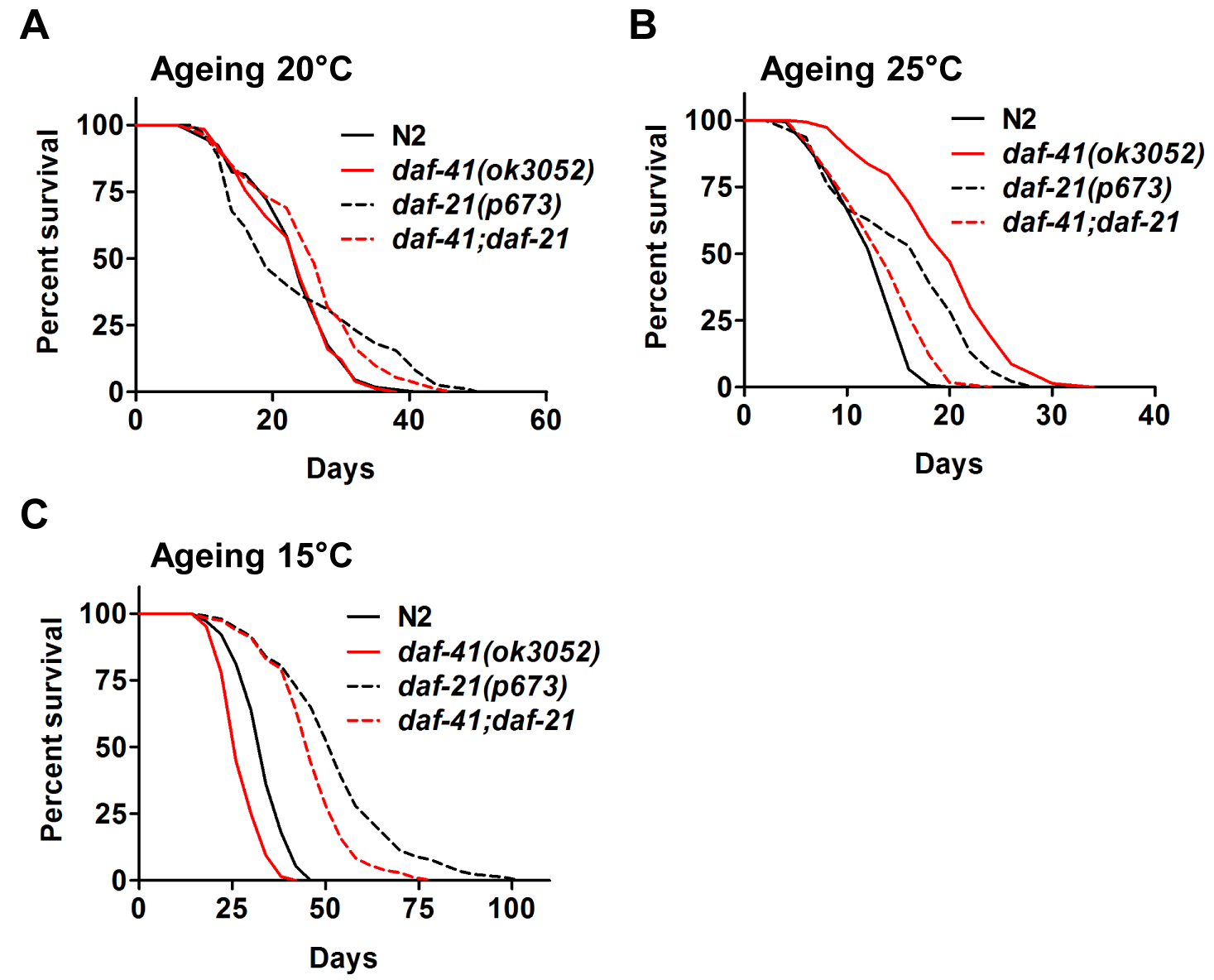

Supplement: S9 Fig — (A) At 20°C, daf-21(p673) and daf-41;daf-21 strains lived slightly longer than N2 (B) At 25°C, daf-21(p673) worms lived slightly longer than N2 but reduced the longevity of daf-41(ok3052). (C) At 15°C, daf-21(p673) and daf-41;daf-21 strains showed extended longevity relative to N2 and daf-41(ok3052) backgrounds. (TIF) [file pgen.1005023.s009.tif]

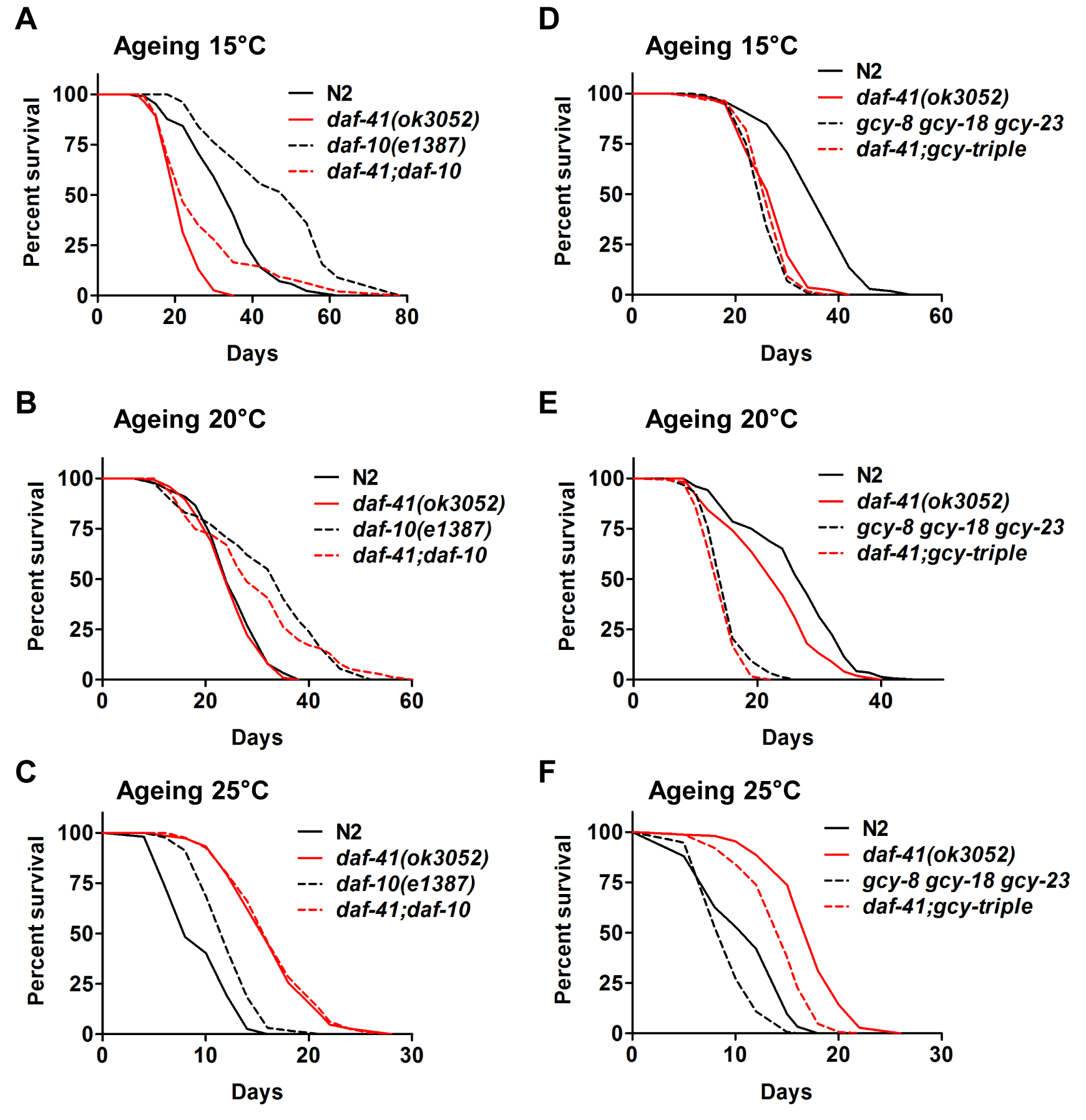

Supplement: S10 Fig — (A-C) daf-10(e1387) worms lived longer than N2 at 15°C, 20°C and 25°C. (A-B) daf-41 regulated lifespan parallel to daf-10 at 15°C and 20°C, (C) daf-10 mutation did not further extend longevity in the daf-41(ok3052) background at 25°C. (D-F) gcy triple mutants [gcy-8(oy44) gcy-18(nj38) gcy-23(nj37)] lived shorter than N2 at 15°C, 20°C and 25°C, but (D) the gcy triple mutant did not further reduce lifespan in the daf-41(ok3052) background at 15°C. (E-F) daf-41 regulated lifespan parallel to the gcy triple mutant at 20°C and 25°C. (TIF) [file pgen.1005023.s010.tif]
