# Supplementary material for: Co-chaperone p23 Regulates C. elegans Lifespan in Response to Temperature
Source: PLoS Genet. 2015 Apr 1;11(4):e1005023. doi: 10.1371/journal.pgen.1005023 (PMC4382338; doi:10.1371/journal.pgen.1005023)
Supplement: S1 Text — Additional details for DiI staining, chemotaxis analysis, oxygen consumption measurement and microscopy analysis are described in S1 Text. (DOCX) [file pgen.1005023.s013.docx]

**Supplemental materials and methods**

**Progeny Measurements**

N2 worms and *daf-41(ok3052)* worms were maintained at 20°C. Single L4 larvae were transferred onto a fresh plate and the number of progeny counted every 2 days until day 8. Twelve worms for each genotype were examined, with 5 biological replicates.

**DiI staining**

N2, *daf-41(ok3052)* and *daf-10(e1387)* worms were stained with 10ng/ml of DiI in M9 for 1hr as described previously [[64](#_ENREF_1)]. Worms were washed with M9 three times after staining and transferred onto a fresh plate to destain for 1hr. Photos were taken under the Texas Red filter.

**Chemotaxis analysis**

Chemotaxis assays were carried out according to [[65](#_ENREF_2)]. All strains were cultivated at 20°C. Synchronized adult worms were washed 3 times with M9 buffer to exclude progeny and bacteria, and transferred onto bacteria-free 10 cm NGM plate. After 1 hour of fasting, 50 worms were transferred into the center of a chemotaxis assay plate. Chemoattractants; isoamyl alcohol, benzaldehyde and 2,4,5-trimethylthiazoline, were diluted a thousand fold in ethanol and 1ul of each chemoattractant was spotted with 1ul of 0.5 mol/l NaN3. One micro litter of ethanol was used as a negative control for chemoattractants.

**Oxygen consumption measurements**

N2 and *daf-41(ok3052)* worms were maintained at 15°C and 20°C respectively. Three hundred mid L4 larvae were collected by a COPAS Biosort worm sorter and cultivated at 15°C, 20°C and 25°C until adult. Adult worms were washed off plates with M9 buffer, and bacteria and progeny removed by repeated washing. O2 consumption at each temperature was measured by O2k-Core and Modular O2k-MultiSensor System (OROBOROS® INSTRUMENTS GmbH, Austria). The experimental detail is described previously [[66](#_ENREF_3)].

**Analysis of egg laying defects**

All strains were cultivated at 20°C and eggs collected by bleaching. Synchronized eggs were seeded onto 3cm OP plates and cultivated at 20°C. When adult worms carried a few eggs, they were transferred singly onto a new 3cm OP plate and allowed to lay eggs for 24 hours. The ratio of mothers with internal hatching was then measured.

**Microscopy analysis**

Strains were maintained at 20°C. We performed temperature shifts from 20°C to 25°C for *muIs109* worms (*daf-16::gfp*) at the L2 stage and observed L4 larvae, and for *drSi13* worms (*hsf-1::gfp*) at the L3 stage and photos were taken with young adult worms. *muIs109* worms were anesthetized with 20mM sodium azide and *drSi13* worms were anaesthetized with 1mM levamisole. Photos were taken in the anterior region for *muIs109* and the posterior tail region for *drSi13*.

**References**

64. Ward S, Thomson N, White JG, Brenner S (1975) Electron microscopical reconstruction of the anterior sensory anatomy of the nematode Caenorhabditis elegans.?2UU. J Comp Neurol 160: 313-337.

65. Hirotsu T, Saeki S, Yamamoto M, Iino Y (2000) The Ras-MAPK pathway is important for olfaction in Caenorhabditis elegans. Nature 404: 289-293.

66. Hench J, Bratic Hench I, Pujol C, Ipsen S, Brodesser S, et al. (2011) A tissue-specific approach to the analysis of metabolic changes in Caenorhabditis elegans. PLoS One 6: e28417.
